# Tracking single-cell evolution using clock-like chromatin accessibility loci

**DOI:** 10.1038/s41587-024-02241-z

**Published:** 2024-05-09

**Authors:** Yu Xiao, Wan Jin, Lingao Ju, Jie Fu, Gang Wang, Mengxue Yu, Fangjin Chen, Kaiyu Qian, Xinghuan Wang, Yi Zhang

**Affiliations:** 1 Department of Urology, Zhongnan Hospital of Wuhan University, Wuhan, China; 2 Department of Biological Repositories, Human Genetic Resources Preservation Center of Hubei Province, Hubei Key Laboratory of Urological Diseases, Zhongnan Hospital of Wuhan University, Wuhan, China; 3 Euler Technology, ZGC Life Sciences Park, Beijing, China; 4 Hong Kong University of Science and Technology, Hong Kong, China; 5 High Performance Computing Center, Peking-Tsinghua College of Life Sciences, Peking University, Beijing, China; 6 Medical Research Institute, Frontier Science Center for Immunology and Metabolism, Taikang Center for Life and Medical Sciences, Wuhan University, Wuhan, China; 7 Wuhan Research Center for Infectious Diseases and Cancer, Chinese Academy of Medical Sciences, Wuhan, China

**Keywords:** Epigenetic memory, DNA methylation, Bioinformatics, Epigenomics

## Abstract

Single-cell chromatin accessibility sequencing (scATAC-seq) reconstructs developmental trajectory by phenotypic similarity. However, inferring the exact developmental trajectory is challenging. Previous studies showed age-associated DNA methylation (DNAm) changes in specific genomic regions, termed clock-like differential methylation loci (ClockDML). Age-associated DNAm could either result from or result in chromatin accessibility changes at ClockDML. As cells undergo mitosis, the heterogeneity of chromatin accessibility on clock-like loci is reduced, providing a measure of mitotic age. In this study, we developed a method, called EpiTrace, that counts the fraction of opened clock-like loci from scATAC-seq data to determine cell age and perform lineage tracing in various cell lineages and animal species. It shows concordance with known developmental hierarchies, correlates well with DNAm-based clocks and is complementary with mutation-based lineage tracing, RNA velocity and stemness predictions. Applying EpiTrace to scATAC-seq data reveals biological insights with clinically relevant implications, ranging from hematopoiesis, organ development, tumor biology and immunity to cortical gyrification.

## Abstract (web summary)

EpiTrace infers cell mitotic age and evolution from single-cell ATAC-seq data.

## Main

Single-cell chromatin accessibility sequencing (scATAC-seq) is a powerful technique for interrogating the epigenomic landscape at single-cell resolution^1^. However, inferring the exact developmental trajectory of cells from scATAC-seq data is challenging. Although tools such as RNA velocity, stemness prediction and metabolic labeling^2–5^ exist for determining cell evolution trajectories on the manifold of phenotypes from single-cell RNA sequencing (scRNA-seq) datasets, no similar methods exist for scATAC-seq. State-of-the-art, similarity-based lineage deduction methods^6^ would be limited when phenotypes are fluidic, such as in dedifferentiation or oncogenesis. On the other hand, mutation-based lineage tracing methods—for example, using mitochondrial single-nucleotide polymorphisms (SNPs)^7–9^, which track the phylogeny of cells over divisions—are highly accurate, yet their temporal resolution is restrained by the low natural mutation rate.

The concept of mitotic age refers to the accumulative counts of mitosis that a cell undergoes after the ground state of cell division or fertilization. The first proposed mitotic (replicational) age biomarker was telomere length, which is genetic^10,11^, and the concept quickly extended to epigenetic replication errors on DNA methylation (DNAm)^12,13^. During DNA replication, epigenetic covalent modifications are not faithfully replicated to the daughter strand, resulting in stochastic DNAm changes. Stochastic DNAm fluctuation has been applied to infer the mean mitotic count of the cell population^14–16^. On the population scale, irreversible, stochastic DNAm changes were thought to be underlying age-associated DNAm changes. A vast number of studies have documented age-associated DNAm changes^17^, including hypomethylation and hypermethylation, in specific genomic regions. We term such genomic regions clock-like differential methylation loci (ClockDML) because their DNAm exhibits timekeeper-like behavior.

The DNAm-based regression model predicts the age of biological samples with extremely high precision in many organisms^18–24^ and correlates with rejuvenation or accelerated aging in various scenarios^25^. A similar age association of DNAm was conserved across mammalian species in homologous genomic regions^26^, suggesting that it is controlled by a defined, possibly functional, molecular mechanism^27^. Interestingly, a predictor model was built to estimate the mitotic age of samples from the DNAm state of a defined set of CpG loci^28,29^, indicating that mitosis is associated with clock-like DNAm changes at specific genomic loci. Introducing population statistics into the DNAm-based age prediction model enabled single-cell age prediction from single-cell methylation sequencing data^30,31^, suggesting that clock-like DNAm changes are not merely a statistical phenomenon at the population scale but also occur at the single-cell level.

Based on the intrinsic link between chromatin accessibility and DNAm^32–37^, we hypothesized that age-dependent DNAm could either result from or result in chromatin accessibility changes at ClockDML. If so, the derived mitotic age of single cells from scATAC-seq data would serve as a powerful tool to delineate developmental trajectory. In theory, mitotic age is a ‘timekeeper’ tracker: the mitotic age of an ancestor cell is lower than that of its progeny, and cells originating earlier in time should show lower mitotic age than those originating later. Such a measure of cell age, if it exists, would provide a precise temporal reference of the cell birth sequence to help delineate the developmental trajectories in a complex organism.

To develop a mitotic age estimator for scATAC-seq data, we determined ClockDML across the human genome and characterized the chromatin accessibility changes in these loci associated with cell mitotic age. The heterogeneity of chromatin accessibility at these loci reduces across cell division. Through genomic synteny mapping, we showed that the age-dependent chromatin accessibility is conserved on these loci across evolution. Such clock-like chromatin accessibility is independent from DNAm. Even in species without active DNAm, clock-like chromatin accessibility exists on these loci. Hence, we term these regions as ‘clock-like loci’. We leveraged this phenomenon to develop a computational framework, called EpiTrace, which infers cell mitotic age from scATAC-seq data by counting the opened fraction of clock-like loci in single cells.

## Results

### Chromatin accessibility enables single-cell age estimation

**Fig. 1:**
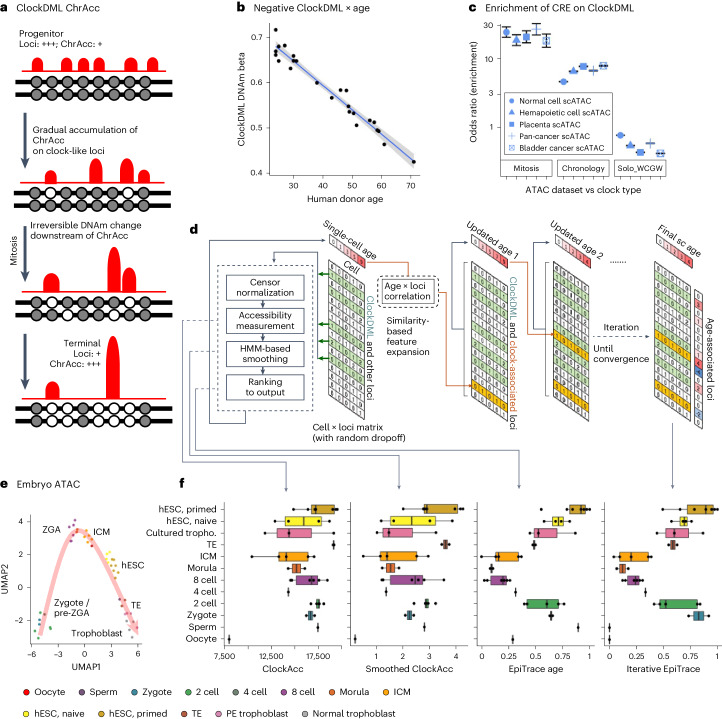
ChrAcc change associated with irreversible DNAm drift on ClockDML enables cell age estimation. **a**, Schematic diagram of the underlying epigenetic mechanism of cell mitotic age tracing using ChrAcc on ClockDML. **b**, Correlation between the DNAm level on G8-group ClockDML and sample age in human PBMCs. 95% CI is shown as a gray area around the linear regression line. *R* = −0.978, *P* < 2.2 × 10^−^^16^. **c**, Enrichment of mitosis-associated ClockDML (Mitosis, size = 1,934 bp), actual age-associated ClockDML (Chronology, size = 58,7801 bp) and solo-WCGW loci (size = 5 Mbp) in each class of ATAC peaks (size = 281 Mbp (TCGA Pan-cancer); 164 Mbp (bladder cancer); 462 Mbp (normal cell); 218 Mbp (placenta); and 295 Mbp (hematopoietic cell)). Two-sided Fisher’s exact test was performed for the expected against observed overlapped region size. The points show the resulting odds ratio of observed size over expected size, and the 95% CI is shown as whiskers. **d**, Overview of the EpiTrace algorithm. **e**, UMAP of the human early embryonic development scATAC dataset. Color indicates the developmental stage of human embryo. **f**, The total chromatin accessibility on ClockDML (ClockAcc), HMM-smoothened ClockAcc and initial and iterative EpiTrace ranking result (EpiTrace age) corresponding to the embryonic dataset. Sample numbers of biologically independent samples: *n* = 1 (Oocyte); 1 (Sperm); 2 (Zygote); 5 (two-cell); 1 (four-cell); 6 (eight-cell); 2 (Morula); 5 (ICM); 4 (Naive hESC); 7 (Primed hESC); 2 (TE); and 3 (Differentiated trophoblast). The upper and lower bounds of boxes show 25% and 75% percentiles of the data. The median of data is shown as the horizontal line in the box. The distribution minima and maxima, defined as farthest data point distanced ≤1.5 IQR from the box bounds, are shown by the whiskers. Correlation R and *P* value: Pearson’s. Tiny *P* values resulting in numerical underflow are shown as ‘<2.2 × 10^−16^’. tropho., trophoblast.

Although the molecular mechanism that generates age-associated DNAm changes is unclear, it is possible that the methylation state of ClockDML might be affected by chromatin accessibility. Alternatively, the methylation state of ClockDML might reversely regulate chromatin accessibility. In either case, the chromatin accessibility on ClockDML could be used to deduce cell age (Fig. 1a). However, the dynamics of chromatin accessibility on ClockDML during aging are currently unknown.

Literature-documented ClockDML^18,25,26,38^ were found mainly with methylation-specific microarrays and, thus, represent only a tiny fraction of possible genome-wide DNAm variation during aging. However, ATAC-seq—and, more so, scATAC-seq—data are too sparse to fully cover these loci. To enable accurate tracking of cell mitotic age by ATAC signal, we determined 126,420 ClockDML in the human genome by bisulfite capture sequencing the CpG island regions of peripheral blood mononuclear cells (PBMCs) from a panel of donors of different ages and determined the correlation coefficient between age and the methylation level (beta) on each locus (Supplementary Table 1: ClockDML). The DNAm status of these ClockDML showed excellent correlation with age in the training cohort (Fig. 1b). Both the general linear model (GLM) and the probability model (TimeSeq)^31^ built upon beta values of our ClockDML predict donor age with good precision in an additional validation cohort of samples (*R* = 0.85 (GLM) / 0.7998 (TimeSeq); Supplementary Fig. 1a–c), indicating that the DNAm status of these loci stably drifts over age.

Functional annotation suggests that ClockDML are enriched in the open, accessible chromatin region of the genome across different cell types and organs^39–41^ (Fig. 1c). In correlation with DNAm status, the fraction of opened ClockDML (hereafter, in short: ClockAcc) shifts in correlation with cell aging (Supplementary Notes and Supplementary Figs. 2–5; GSE74912 (ref. ^42^), GSE89895 (ref. ^43^) and GSE179606 (ref. ^44^)). We established an algorithm, called EpiTrace, that predicts sample age by counting the fraction of opened ClockDML in bulk ATAC-seq datasets (Supplementary Notes). Validation experiments using bulk ATAC-seq of FACS-sorted blood cells, induced pluripotent stem cell (iPSC) induction experiments and native immune cells showed that EpiTrace accurately predicted sample age in concordance with known developmental trajectories (Supplementary Notes and Supplementary Figs. 2–5). We then adopted the algorithm for scATAC-seq data. In brief, a reference ClockDML set was provided to the algorithm. ClockAcc, the total chromatin accessibility on this reference ClockDML set, is measured for each cell. The measurement was performed using a hidden Markov model (HMM)-mediated diffusion-smoothing approach, borrowing information from similar single cells to reduce noise in the single-cell measurement: cells were clustered via correlation of top variable ATAC peaks to form a cell‒cell similarity matrix, which was then used for diffusion-regression iterations of ClockAcc until convergence. After iteration, the regularized and smoothened ClockAcc of each single cell were then ranked. Such rank denotes the relative mitotic cell age. To overcome sampling sparseness in scATAC-seq, we reasoned that it might be unnecessary for age-dependent chromatin accessibility (ChrAcc) to always be accompanied by DNAm changes (Supplementary Notes). Thus, we perform stepwise iterations by extracting additional open regions with a high correlation coefficient with estimated single-cell age and then include them together with the reference loci to form a new set of reference clock-like loci for the next round of analysis until the age prediction converges (Fig. 1d). During the computation of EpiTrace algorithm, known sample age information is not required. The algorithm simply leverages the fact that heterogeneity of given reference ClockDML reduces during cell replication and then uses such information as an intermediate tool variable to infer cell age.

We first validated the EpiTrace algorithm on in vitro models. ChrAcc in single mouse cells was profiled with the simultaneous high-throughput ATAC and RNA expression with sequencing (SHARE-seq) assay (Supplementary Fig. 6), and DNAm age (in batch) was determined by DNAm sequencing (Supplementary Fig. 1d–g). In asynchronized immortal mouse embryonic fibroblast (MEF) cells, progression in the cell cycle results in a reduction in EpiTrace-predicted age (Supplementary Fig. 7), suggesting that EpiTrace tracks an epigenomic modification that dilutes during genome replication (as newly synthesized copy of genome emerges). In primary MEF (pMEF) cells, this phenomenon persists (Supplementary Fig. 8a,b,d,h). However, as the cells were passaged in vitro, the EpiTrace age stably increased (Supplementary Fig. 8b,i). Such a mitosis age-dependent increase in EpiTrace age overwhelms the genome replication-mediated dilution effect (Supplementary Fig. 8) and correlates well with DNAm-based age prediction of the same batch of samples (Supplementary Fig. 9). Finally, for cells that are pharmacologically blocked in a specific cell cycle (GSE65360 (ref. ^1^)), EpiTrace age increases from G1 to S and G2/M phase (Supplementary Fig. 10), suggesting that accumulation of error during copying of epigenomic modification to the newly synthesized copy of genome results in an increase in EpiTrace age prediction over mitosis. In large in vivo single-cell datasets without cell phase synchronization, the cell cycle had little effect on EpiTrace age prediction (Supplementary Fig. 11; GSE163579). Together, these data indicate that EpiTrace reports mitosis age.

As a proof of concept, we gathered ATAC data from various studies of early human embryonic development from gametes to blastula^19,45^ (Fig. 1e and Supplementary Fig. 12; PRJNA494280 and PRJNA394846), which were generated from only a few cells each, and subjected them to EpiTrace analysis without batch correction (Fig. 1f). The total ClockAcc in sample positively correlates with known cell mitotic age. Although the initial EpiTrace age prediction is noisy, iterative optimization improved the signal-to-noise ratio to draw a biologically plausible trajectory of age resetting during early embryonic development: starting from zygote, cell mitotic age gradually reduces to near ground state at the time of zygotic genome activation (ZGA) at morula, before its rebound in inner cell mass (ICM), trophectoderm (TE) and embryonic stem cell (ESC).

### Inferring cell age across cell types and animal species

For many cell types and animal species, ClockDML have not been experimentally determined. The fact that ClockDML derived from human PBMCs could be used to predict the sample age not only of human blood cells but also of cells of the non-hematopoietic lineage (Fig. 1 and Supplementary Fig. 5) suggests that clock-like ChrAcc on the ClockDML genomic region might be universal across cell lineages. To test whether we could extend known ClockDML to other species or cell types for EpiTrace prediction, we mapped human ClockDML to the mouse genome using genomic synteny and computed EpiTrace age for the mouse scATAC-seq dataset using mouse ClockDML or ‘human-guided’ clock-like loci (Supplementary Fig. 13a). We found that the EpiTrace prediction results using the reference ‘human-guided’ clock-like loci closely approximated the prediction results using the reference mouse ClockDML (*R* = 0.81; Supplementary Fig. 13b; GSE137115 (ref. ^46^)).

To further validate the concordance between EpiTrace prediction starting from different reference loci, we tested a mouse scATAC-seq dataset of T cells under chronic or acute virus infection (Supplementary Fig. 14a; GSE164978 (ref. ^47^)). EpiTrace age prediction using the mouse reference ClockDML agrees with the known developmental trajectory of these immune cells (Supplementary Fig. 14b). In concordance with their tissue of origin, clock-like loci inferred from genomic synteny of human PBMC ClockDML overlap with known immune cell exhaustion genes, such as *Pdcd1*, *Havcr2*, *Tox* and *Eomes*, whereas mouse ClockDML^48^ (derived from pan-body DNAm interrogation) do not (Supplementary Fig. 14c). However, single-cell age inferred by EpiTrace with mouse ClockDML as reference correlates well with that inferred with ‘human-guided’ clock-like loci as reference (Supplementary Fig. 14d). The association of ATAC peak chromatin accessibility and single-cell ages from the two predictions shows extremely high concordance (*R* = 0.92; Supplementary Fig. 14e), with the identification of many known immune exhaustion genes being positively correlated with cell age (Supplementary Fig. 14e). Such correlation is not dependent on whether the loci are previously overlapping with a reference clock-like loci (Supplementary Fig. 14f). Furthermore, peaks overlapping with both ‘human-guided’ clock-like loci and mouse ClockDML showed the greatest age-dependent ChrAcc shift (Supplementary Fig. 14g). These results indicate that EpiTrace can use ClockDML from different tissues of origin to predict single-cell age, even in a cross-species scenario.

**Fig. 2:**
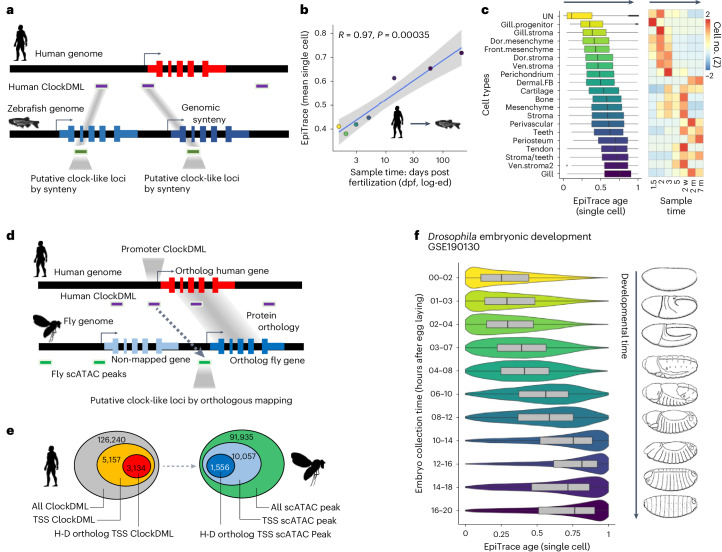
Mapping ClockDML orthologous genomic regions across species facilitates single-cell age estimation using ChrAcc. **a**, Schematic of the experiment. Human ClockDML are mapped to the zebrafish genome by homology to produce ‘human-guided reference clock-like loci’ and are then used to infer zebrafish neural crest cell mitotic age. Because the data were provided as a one-hot matrix, we adopted the bulk ATAC-like algorithm output. **b**, Linear regression of predicted mean mitotic age (*y* axis) against log-transformed (log-ed) days post-fertilization (dpf) of the sample (*x* axis). 95% CI is shown as a gray area around the linear regression line. **c**, Single-cell mitotic age of each cell type (left) and cell-type-specific prevalence (Z-scaled, Z) in samples of different ages (right). Sample numbers of biological independent cells: *n* = 11,234 (UN, undefined); 2,223 (gill progenitor); 2,408 (gill stroma); 2,877 (dorsal mesenchyme); 1,363 (frontal mesenchyme); 2,060 (dorsal stroma); 1,412 (ventral stroma); 2,373 (perichondrium); 265 (dermal FB); 3,252 (cartilage); 1,656 (bone); 1,716 (mesenchyme); 1,262 (stroma); 382 (perivascular); 623 (teeth); 2,123 (periosteum); 2,825 (perichondrium); 1,329 (stroma/teeth); 1,009 (ventral stroma 2); and 7,127 (gill). **d**, Schematic of defining putative counterparts of human clock genomic loci in the *Drosophila* genome. Human ClockDML falling within ±100 bp of the gene transcription start site (TSS) were defined as ‘Promoter ClockDML’. For human genes that simultaneously have a promoter ClockDML and one or more *Drosophila* ortholog gene(s), we define any *Drosophila* scATAC peaks falling within ±100 bp of the TSSs of these *Drosophila* orthologs as putative clock-like genomic loci. These loci were subsequently used for EpiTrace analysis in the *Drosophila* dataset. **e**, Diagram showing the number of ClockDML and scATAC falling in each category. H-D: human-drosophila pair. **f**, EpiTrace age of *Drosophila* embryonic development time series samples taken every 2 hours after egg laying (GSE190130 (ref. ^53^)). Corresponding embryo sketches are shown on the right. Sample number of biological independent cells: *n* = 20,000 for each time slot. For box plots, the upper and lower bounds of boxes show 25% and 75% percentiles of the data. The median of data is shown as the horizontal line in the box. The distribution minima and maxima, defined as farthest data point distanced ≤1.5 IQR from the box bounds, are shown by the whiskers. The violin plot shows the empirically estimated density distribution of datam. m, months; w, weeks.

We then mapped human ClockDML to the zebrafish genome using a similar synteny-guided approach (Fig. 2a) and tested EpiTrace prediction on a zebrafish scATAC-seq dataset spanning from fertilization to the adult stage (GSE178969 (ref. ^49^)) using this ‘human-guided’ clock-like loci as reference. The mean EpiTrace age prediction from each stage closely approximated the known sample age (*R* = 0.97; Fig. 2b). For each single-cell type, the EpiTrace prediction closely assembles their time of emergence (Fig. 2c). Similar results were obtained for ‘mouse-guided’ clock-like loci (Supplementary Fig. 13c; GSE152423 (ref. ^50^)).

For many animal species, active DNAm is not present in the genome. For example, the *Drosophila melanogaster* genome has less than 1% of CpG being methylated^51,52^. We hypothesized that if clock-like chromatin accessibility is a universal phenomenon in ClockDML-homologous genomic regions, then identification of ClockDML-homologous genomic regions in such species might be sufficient for age prediction using EpiTrace.

Because these animal species are evolutionarily too distant from humans, only gene-level orthologous relationships could be reliably identified between their genomes and humans. To overcome this problem, we used an orthology-guided approach to first identify human–animal orthologous genes whose promoters encompassed ClockDML in the human genome and then to identify the corresponding promoter genomic loci in the distant animal genome (Fig. 2d). For the *D. melanogaster* genome, we identified 1,556 such loci (Fig. 2e). We then used this as ‘human-guided’ clock-like loci in the *Drosophila* genome for reference in EpiTrace age prediction in a *Drosophila* embryonic development scATAC-seq dataset. The prediction result showed high concordance with the known sampling time (Fig. 2f; GSE190130 (ref. ^53^)).

### ChrAcc change is upstream of DNAm shift on ClockDML

*Drosophila* is an invertebrate species that lacks canonical DNA methyltransferase. Only 0.4% (1 hour post-fertilization (hpf)) to 0.1% (12 hpf) of cytosine in the *Drosophila* genome was methylated^52^ compared to 6–8% in humans, and most *Drosophila* methylated C was CpT/CpA. The fact that EpiTrace can work on the *Drosophila* genome suggests that clock-like chromatin accessibility might be independent of clock-like DNA methylation. To validate this model, we first tracked ChrAcc and DNAm on the same DNA molecule using the human embryonic development single-cell chromatin overall omic-scale landscape sequencing (scCOOL-seq) dataset (Supplementary Fig. 15; GSE100272 (ref. ^54^)) and a long-read nanopore sequencing of nucleosome occupancy and methylome (nanoNOME) dataset (Supplementary Fig. 16; GSE183760 (ref. ^55^)). In both datasets, ChrAcc shifts before ClockDML DNAm changes on the same molecule, indicating that clock-like DNAm is not necessary for clock-like ChrAcc.

We then performed forced transcriptional activation around ClockDML to test whether changes in ChrAcc would influence DNAm in these regions. We transfected single guide RNA (sgRNA) lentivirus targeting human G8-group ClockDML loci (shown in Fig. 1b) into HEK293 cells stably expressing the dCas9–p300 transactivator (Supplementary Fig. 17a). Gain of ChrAcc around these loci results in DNA hypomethylation on neighboring ClockDML (Supplementary Fig. 17b,c), indicating that clock-like DNAm could be driven by ChrAcc shift.

In another conceptually similar scenario, we measured the DNAm changes under forced transcription activation around mouse *Sox1* loci and found them to linearly correlate with the age-dependent DNAm shift coefficient of corresponding loci in the human genome (Supplementary Fig. 18; PRJNA490128 (ref. ^56^)). Hence, changes of ChrAcc on ClockDML are sufficient to drive clock-like differential DNAm.

To validate whether changes in DNAm would affect ChrAcc on ClockDML, we tracked ChrAcc in a dataset where forced DNAm was performed with a ZNF-DNMT3A artificial methylator (Supplementary Fig. 19a; GSE102395 (ref. ^57^)). Although ZNF-DNMT3A induction results in irreversible DNAm on ClockDML around its binding site (Supplementary Fig. 19b,e), it does not change the overall ChrAcc on these loci (Supplementary Fig. 19c,d; GSE102395 (ref. ^57^) and GSE103590 (ref. ^58^)) nor does it change the EpiTrace age on these cells (Supplementary Fig. 19f,g; GSE102395 (ref. ^57^) and GSE103590 (ref. ^58^)).

Together, these data indicate that clock-like ChrAcc occurs upstream of the DNAm shift on ClockDML. In animals without active DNAm, genomic region exhibiting clock-like ChrAcc could also be identified. In other words, clock-like ChrAcc is an innate property on the clock-like loci, which usually harbor ClockDML. Furthermore, clock-like ChrAcc is independent from DNAm.

### The reversal of epigenetic age during iPSC induction

**Fig. 3:**
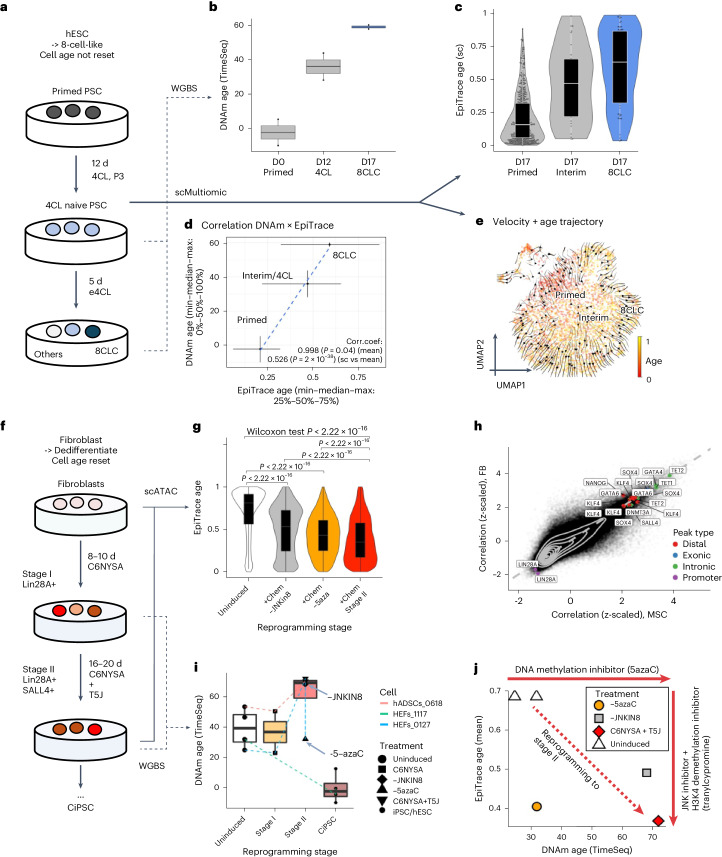
Inferring single-cell age reversal in iPSC induction with EpiTrace. **a**, Schematic overview of the in vitro chemical induction of human pluripotent stem cells (‘Primed’) back to 8-cell like cell (8CLC) state, through serially culturing in 4-cell-like medium (4CL, three passages (P3)) and the enhanced 4CL-medium (e4CL). **b**, DNAm age of D0 (day 0, Primed) and D12 (day 12, 4CL) cultures and sorted 8CLCs from D17 (day 17) culture, from WGBS data. *n* = 2 independent biological repeats in each group. **c**, Single-cell age estimated with EpiTrace from the D17 scMultiomic dataset. Sample numbers of biologically independent cells: *n* = 483 (primed); 33 (interim); and 61 (8CLC). **d**, Correlation of inferred age from DNAm (whiskers denote minimum/maximum, central point denotes median value, *y* axis) or single-cell EpiTrace age (whiskers denote 25%/75%, central point denotes median value, *x* axis) from the same set of cells. Correlation R and *P* value: Pearson’s. Sample numbers of WGBS and single cells were as in **b** and **c**. **e**, UMAP of scMultiomic-sequenced D17 culture with single-cell evolution trajectories built with kernels combining EpiTrace age and RNA velocity information. **f**, Schematic overview of the in vitro chemical induction of human adult fibroblasts toward chemically induced pluripotent stem cells (CiPSC). Both the uninduced and intermediate stage II cultures were sequenced by scATAC. 5-azaC, 5-azacytidine; C6NYSA, combination of CHIR99021, 616452, TTNPB, Y27632, SAG and ABT869; hADSC, human adipose stromal cell (mesenchymal stromal cell); HEF, human embryonic fibroblast; JNKIN8, c-Jun N-terminal kinase inhibitor; T5J, tranylcypromine, JNKIN8 and 5-azaC. **g**, Single-cell age estimated with EpiTrace from **f**. The induced cultures were either subjected to the full induction paradigm (+Chem: C6NYSA + T5J) or had 5-azaC or JNKin8 removed (−5aza, −JNKin8). Sample numbers of biologically independent cells: *n* = 8,826 (uninduced); 4,667 (+Chem, −JNKin8); 10,257 (+Chem, −5aza); and 8,671 (+Chem stage II). Statistical comparisons are shown between groups by two-sided Wilcoxon test. **h**, Correlation coefficient between the ChrAcc on each ATAC peak and EpiTrace age estimated from the MSC experiment (*x* axis) or the FB experiment (*y* axis). Peaks of interest are labeled, colored by their genomic location class. **i**, Prediction of sample age by DNAm from WGBS data of chemical induction of iPSCs. Chemical reprogramming induces genome-wide demethylation and an increase in DNAm age, as reported previously^31^, whereas the addition of 5-azaC globally reduces DNAm to increase DNAm age. Removal of 5-azaC blocks DNAm age from increasing. Sample numbers of biologically independent samples: *n* = 4 (uninduced); 2 (C6NYSA); 1 (−JNKIN8); 1 (−5azaC); 2 (C6NYSA + T5J); and 4 (iPSC/hESC). **j**, Scatter plot of WGBS DNAm age (*x* axis) and mean single-cell EpiTrace age (*y* axis) of the same sample. For box plots, the upper and lower bounds of boxes show 25% and 75% percentiles of the data. The median of data is shown as the horizontal line in the box. The distribution minima and maxima, defined as farthest data point distanced ≤1.5 IQR from the box bounds, are shown by the whiskers. The violin plot shows the empirically estimated density distribution of data. Corr.coef., correlation coefficient.

We tested EpiTrace on a single-cell multiome (scMultiomic) sequencing dataset (CNP0001454 (ref. ^59^)) of primed human embryonic stem cell (‘Primed’ hESC) cultures undergoing chemical reprogramming through a ‘4CL naive PSC’ state toward an eight-cell-like (‘8CL’) state (Fig. 3a), measured EpiTrace age in single cells and compared the EpiTrace prediction with biological age predicted by whole-genome bisulfite sequencing (WGBS) of the same cultures^59^. Both DNAm-based prediction of sample age (Fig. 3b) and single-cell age predicted by EpiTrace (Fig. 3c) suggest that mitotic age increases as cells undergo transformation, with single-cell age gradually increasing across the evolutionary trajectory toward the 8CL state (Supplementary Fig. 20). Furthermore, the biological age estimation of DNAm and EpiTrace was precisely correlated (correlation coefficient of mean single-cell (sc) EpiTrace age × mean DNAm age: 0.998 (*P* = 0.04); scEpiTrace age × mean DNAm age: 0.526 (*P* = 1.9 × 10^−38^)) (Fig. 3d). While RNA velocity projections on these cells showed erroneous evolution trajectories rooted at single cells of a differentiated state (Supplementary Fig. 21), combining RNA velocity and EpiTrace age of the same cell results in more biologically plausible evolution trajectories with the primed hESC as the root of all other cells (Fig. 3e and Supplementary Fig. 21). These results suggest that ChrAcc on ClockDML predicts single-cell biological age at least as well as the DNAm-based age estimator, even in an age-reverse scenario.

We measured EpiTrace age in an additional scATAC-seq dataset of cells undergoing the early stages of chemical reprogramming from differentiated endodermal cells (fibroblast (FB) or mesenchymal stem cell (MSC)) toward iPSCs (Fig. 3f; GSE178324 (ref. ^60^)) and compared them with WGBS-predicted ages from the same study. EpiTrace age prediction of single cells significantly decreased at stage II compared to the uninduced state (Fig. 3g), indicating that these cells are ‘rejuvenated’ as expected. Compared to uninduced cells, the EpiTrace age of stage II reprogrammed C6NYSA + T5J cells is significantly rejuvenated (decreased). Removal of 5-azaC from the treatment result only slightly impairs the ChrAcc age rejuvenation as reflected by EpiTrace age. On the contrary, removal of the JNK inhibitor from the treatment resulted in more significant impairment of rejuvenation (Fig. 3g). The age–peak association from the MSC reprogramming experiment is highly similar to that from the FB reprogramming experiment. (Fig. 3h). These results suggest relevance between the observed mitotic age resetting and cell fate reprogramming.

The DNAm-predicted age of cells during the chemical induction procedure shows that the biological age first increases at induction stage II (Fig. 3i; GSE178966 (ref. ^60^)) before decreasing to near zero in the pluripotent state. Removal of 5-azaC from the induction formula blocks the DNAm age increase at stage II, indicating that the apparent DNAm age increase is a result of global DNA demethylation^31^. Similar to our previous observations, comparing the DNAm age and EpiTrace age prediction of the same cell sets suggests that ChrAcc on ClockDML is independent from ClockDML DNAm change (Fig. 3j).

### Epigenetic age determines future cell expansion potential

**Fig. 4:**
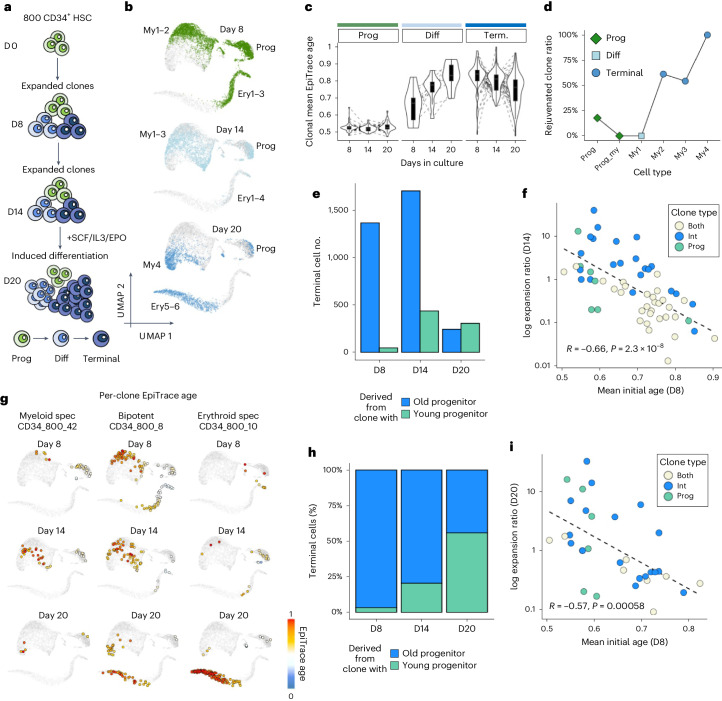
Single-cell age estimation revealed that epigenomic age determines clonal expansion potential. **a**, Schematic of the experiment. CD34^+^ HSCs were used in the in vitro expansion/differentiation experiment. Cells were first expanded to D8 (CD34_500) or D14 (CD34_800) and then differentiated by SCF, IL-3 and EPO until D20. Mitochondrial mutations from the scATAC experiment were used for tracking cells derived from similar clones. Cell phenotypes were determined by the scATAC profile. **b**, Cells from experiments performed on D8, D14 and D20, showing a gradual transition toward terminally differentiated myeloid (my4) and erythroid (ery6) cells. **c**, Tracking the mean EpiTrace age of each myeloid cell clone at each timepoint. Sample numbers of independent biological clones: *n* = 35 (Prog D8–D20); 10 (Diff D8–D20); and 67 (Terminal D8–D20). **d**, Ratio of rejuvenated (clone age decrease over time) clones in all clones for the myeloid cells. The terminally differentiated cells are dominated by rejuvenated clones. **e**, Number of terminal myeloid cells derived from young proliferator clones (mean initial clonal EpiTrace age < 0.7) and old proliferator clones (mean initial clonal EpiTrace age ≥ 0.7) at three timepoints. **f**, Scatter plot of the log clonal expansion ratio on D14 (*y* axis) compared to the mean initial clonal EpiTrace age of the same clone (*x* axis). Clonal types are color-labeled. **g**, EpiTrace age (color) of the single cells derived from a similar clone. Three clones with different fates are shown for example. The CD34_800_42 clone was a myeloid-specific clone that generated only myeloid cells. The CD34_800_8 clone was a bipotent clone that generated both myeloid and erythroid decedents. The CD34_800_10 clone was an erythroid-specific clone that generated predominantly erythroid cells. **h**, Relative contribution of young proliferator clones (mean initial clonal EpiTrace < 0.7) and old proliferator clones (mean initial clonal EpiTrace age ≥ 0.7) in the terminal myeloid cell population at three timepoints. **i**, Scatter plot of the log clonal expansion ratio on D20 (*y* axis) compared to the mean initial clonal EpiTrace age of the same clone (*x* axis). Clonal types are color-labeled. Correlation statistics (*R* and *P* value): Pearson’s. Group statistics: *t*-test, two-sided. For box plots, the upper and lower bounds of boxes show 25% and 75% percentiles of the data. The median of data is shown as the horizontal line in the box. The distribution minima and maxima, defined as farthest data point distanced ≤1.5 IQR from the box bounds, are shown by the whiskers. The violin plot shows the empirically estimated density distribution of data.

To test EpiTrace age estimation in genetically defined cell lineages, we took advantage of a mitochondria-enhanced scATAC-seq dataset (GSE142745 (ref. ^8^)) of cultured CD34 hematopoietic stem cells (HSCs) that underwent in vitro expansion for 14 d before being forced into differentiation under SCF/IL3/EPO toward myeloid/erythroid lineages for an additional 6 d (Fig. 4a). These cells were sequenced at day 8 (D8), day 14 (D14) and day 20 (D20). Cells were clustered by their transcriptomic (scRNA) phenotype as progenitor (Prog) cells, differentiated (Diff) cells or terminally (Terminal) differentiated cells, which gradually emerged over days in culture (Fig. 4b and Supplementary Fig. 22). Furthermore, they were segregated into lineages (clones) arising from the same progenitor by mitochondrial single-nucleotide variant (SNV).

We used EpiTrace to predict mitosis age in these cells, separately for myeloid lineage and erythroid lineage cells. Because the single-cell age prediction by EpiTrace could be affected by highly biased cell composition (Supplementary Fig. 23), we selected a relatively balanced CD34_800 dataset for erythroid lineage cell age prediction. Both CD34_500 and CD34_800 datasets were used for myeloid lineage cell age prediction.

The age prediction shows high concordance with known sampling days across cell types and enables tracking the mitosis age of individual cells derived from the same clone (Fig. 4g). For the myeloid lineage, the EpiTrace age of cells from the same clone increased from progenitor to terminal myeloid cells (Fig. 4c,d). Forced differentiation increased the age of differentiated cells as expected but decreased the age of terminal cells (Fig. 4c,d). To explore this phenomenon in depth, we classified clones according to the relative age change between days of culture (Supplementary Fig. 24): clones that exhibited an age increase from D8 to D14 in a cluster were classified as ‘Aged’, and those that exhibited an age decrease from D8 to D14 were classified as ‘Rejuvenated’. Although most progenitor and differentiated cells show clonal aging during induction, clonal rejuvenation dominates the terminally differentiated clusters (Fig. 4c,d). The proportion of clones showing a rejuvenation increase in terminal cells (Fig. 4d), in correlation with their differentiation state, suggests that these terminally differentiated cells were derived from younger hematopoietic progenitors instead of existing intermediate differentiated cells.

To validate this hypothesis, we analyzed the expansion capability of different cell clones, which processes different types of proliferating cells, including progenitor (Prog) cells and intermediate (Int) differentiated cells, from the CD34_800 experiment (which was sequenced on all three timepoints). We first classified the cell clones according to their cell composition on D8 (the first timepoint): at this time, clones with only Prog cells but no Int cells were classified as ‘Prog-only’; clones with only Int cells but no Prog cells were classified as ‘Int-only’; and clones with both Int and Prog cells were classified as ‘Both’ (Supplementary Fig. 25a). The mean EpiTrace age of the clones at the initial timepoint was measured as mean EpiTrace age of cells from D8 (Supplementary Fig. 25b,d). We then tracked their clonal derivatives at the next timepoints (D14 and D20) to see if a clone was expanded (defined as an increased terminally differentiated cell number at later timepoints compared to D8). For each expanded clone, we calculated the clonal expansion ratio, defined as the increased number of terminally differentiated cells divided by the total cell number on D8.

At both D14 and D20, the log clonal expansion ratio was inversely correlated with the initial EpiTrace age of the clone (Fig. 4f,i and Supplementary Fig. 25c,e): the correlation between the log clonal expansion ratio and initial clonal age was R = −0.66 (*P* = 2.3 × 10^−8^) for D14 and R = −0.57 (*P* = 0.00058) for D20. Although Int-only clones expanded better than Prog-only clones at earlier timepoints (Supplementary Fig. 25c), the Prog-only clones caught up at the latter timepoint and showed improved expansion potential (Supplementary Fig. 25e).

We then re-classified the clones by their mean clonal age at D8 into ‘young progenitor-derived clones’ (defined as the mean EpiTrace age < 0.7) or ‘old progenitor-derived clones’ (defined as the mean EpiTrace age ≥ 0.7). The number of terminal cells derived from young clones steadily increased during the stimulation timecourse, outnumbering the terminal cells derived from old clones on D20 (Fig. 4e). As a result, the relative contribution of terminal cells from young clones steadily increased during the stimulation timecourse (Fig. 4h and Supplementary Fig. 25f,g), explaining the observed decrease in terminal cell EpiTrace age (Fig. 4c,d).

Combining the observations, we conclude that the clonal expansion potential is better explained by clonal epigenetic age instead of the initial phenotype of proliferating cells in the clone. Interestingly, the initial clonal age in clones with both Prog and Int cells was significantly older than that in Prog-only or Int-only clones (Supplementary Fig. 25b,d). These clones expanded the least at both timepoints (Fig. 4f,i and Supplementary Fig. 25c,e). This result indicates that cells in these clones, although phenotypically classified as capable of proliferation, are at the end of their expansion potential.

Together, these results support the model that, during in vitro HSC-stimulated expansion, terminally differentiated cells are preferentially derived from younger progenitors. In other words, younger hematopoietic progenitor cells are much more capable of expansion and differentiation. In the seminal study in which Hayflick determined the in vitro passage limit of cultured cells^61^, he co-cultured 46,XX and 46,XY cells with different in vitro passage numbers together. By counting the karyotypes of cells in the final passage population, he found that the ‘younger’ cells with less starting passage number always dominated the final passage population. Our current experiment is, by design, similar to Hayflick’s original experiment by using a genetic marker, mitochondrial mutation, to track each clone. By measuring the ‘clonal age’ of these single cells, EpiTrace derived a quantitative measure of future expansion potential against the current age of the clone. A pioneering study showed that the genome-wide DNAm level decreases during cell culture passage^62^. This phenomenon was later used to propose a method to infer Hayflick’s limit for individual cell lines^63^. This result provided experimental evidence for the pioneering theoretical works.

### Elucidating T cell markers underlying anti-PD1 response

**Fig. 5:**
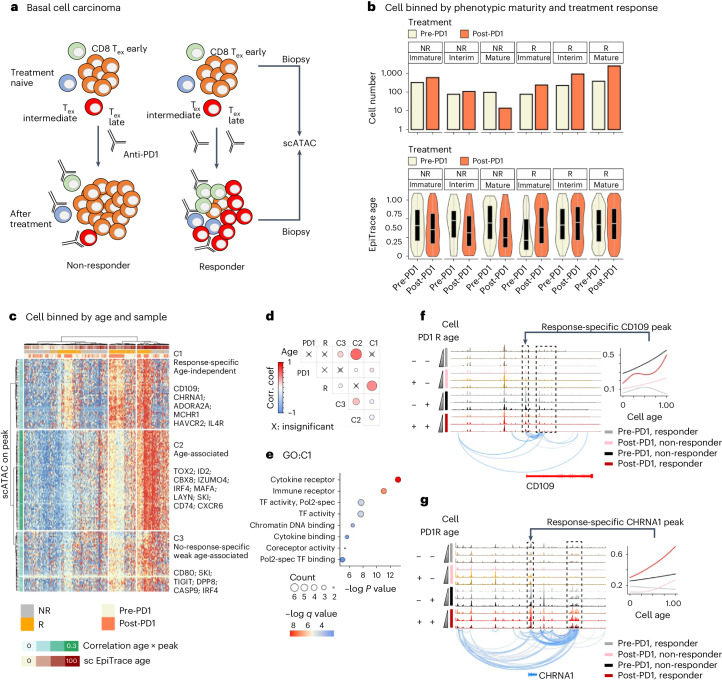
Single-cell age estimation facilitates the discovery of molecular markers of peripheral influx T cells underlying the anti-PD1 response. **a**, Schematic overview of the experiment. Biopsies were taken from patients with basal cell carcinoma before (pre) and after (post) anti-PD1 treatment and subjected to scATAC-seq. **b**, Cell number (above) and EpiTrace age (below) of T_ex_ cells, separated by treatment response (R: responder; NR: non-responder) and T cell phenotypic maturity (Immature/Interim/Mature). Sample numbers of independent biological cells: *n* = 322 (NR group, Immature cell, Pre-PD1); 596 (NR group, Immature cell, Post-PD1); 77 (NR group, Interim cell, Pre-PD1); 108 (NR group, Interim cell, Post-PD1); 97 (NR group, Mature cell, Pre-PD1); 14 (NR group, Mature cell, Post-PD1); 77 (R group, Immature cell, Pre-PD1); 237 (R group, Immature cell, Post-PD1); 222 (R group, Interim cell, Pre-PD1); 923 (R group, Interim cell, Post-PD1); 378 (R group, Mature cell, Pre-PD1); and 2,452 (R group, Mature cell, Post-PD1). **c**, Heatmap showing scATAC peak activity in pseudobulk single cells grouped by phenotype (R/NR), sampling time (pre-PD1 or post-anti-PD1) and EpiTrace age. Correlations between peak activity and EpiTrace age are shown on the left. Peaks were clustered according to their activity profile into response-specific, non-response-specific and age-associated clusters. **d**, Correlation coefficient between clusters of peaks and treatment (PD1: pre/post = 0/1), response (R: NR/R = 0/1) and cell age (Age). Non-significant correlations are labeled with ‘X’. **e**, GO enrichment of the C1 cluster peaks as in **c**. Enrichment was tested by one-sided Fisher’s exact test. −log*P* values were adjusted by multiple comparison. **f**, ChrAcc (top) and cross-correlation between peaks (bottom) of *CD109* loci from pseudobulk single cells grouped with phenotype (R/NR), sample (pre-PD1 or post-anti-PD1) and EpiTrace age (Young/Interim/Mature). The association of the *CD109* promoter ChrAcc across age is shown in the right panel. **g**, ChrAcc (top) and cross-correlation between peaks (bottom) of *CHRNA1* loci from pseudobulk single cells grouped as in **f**. The association of the *CHRNA1* promoter ChrAcc across age is shown in the right panel. Correlation test: Pearson’s. For box plots, the upper and lower bounds of boxes show 25% and 75% percentiles of the data. The median of data is shown as the horizontal line in the box. The distribution minima and maxima, defined as farthest data point distanced ≤1.5 IQR from the box bounds, are shown by the whiskers. The violin plot shows the empirically estimated density distribution of data. Corr.coef., correlation coefficient.

The CD34 dataset demonstrated above is based on an ideal in vitro scenario with cells cultured in an isolated dish. The cultures start at a similar starting point. They proliferate and die in the dish, without exchange with the external environment. To test EpiTrace in a more complex cell population in an in vivo setting, with possible influx, efflux and proliferation, we applied EpiTrace to an scATAC-seq dataset comprising biopsies from basal cell carcinoma pre-anti-PD1 and post-anti-PD1 treatment (Fig. 5a; GSE129785 (ref. ^64^)). After anti-PD1 treatment, cytotoxic T cells with exhaustion markers are significantly increased in anti-PD1 responders (R) but not in non-responders (NR). More immature exhausted T (T_ex_) cells were present in non-responders, and this phenomenon was exaggerated after anti-PD1 treatment. However, overall maturity did not change in responders. The EpiTrace age of interim and mature T_ex_ cells in responders did not change after the anti-PD1 treatment, suggesting that the increased cell number might not be solely due to local proliferation of pre-anti-PD1 mature T_ex_ cells (Fig. 5b).

New post-anti-PD1 mature T_ex_ cells could be derived either from pre-anti-PD1 immature T_ex_ cells or from the influx of peripheral T cells. To test these alternatives, we performed a correlation of ChrAcc on T_ex_ differentially expressed peaks and cell age. Hierarchical clustering of the peak openness of pseudobulk cells from similar age and phenotype segregated peaks into three clusters: C1: response-specific and age-independent; C2: response-irrelevant and age-associated; and C3: non-response-specific peaks that were weakly associated with age (Fig. 5c,d). Interestingly, known markers of activated (*TIGIT*, *LAYN* and *HAVCR2*) and tumor-reactive T cells (*ENTPD1*) were segregated into different clusters. T cell markers (*TOX2*, *ID2* and *MAFA*) and tissue-resident marker *CXCR6* belong to the group of scATAC peaks that are mainly associated with age but are not associated with PD1 response (Fig. 5c,d). The anti-PD1 response is not associated with cell age but, instead, with C1 peak expression. In contrast, cell age is associated with C2/C3 peaks, which are related to no response under anti-PD1. Gene Ontology (GO) enrichment of this C1 cluster, in contrast to C2/C3 cluster genes, showed particular enrichment in the ‘cytokine receptor’ and ‘immune receptor’ pathways (Fig. 5e and Supplementary Fig. 26), highlighting genes such as *IL4R*, *CD74*, *IFNGR2* and *IFNAR2*, which might be implicated in the anti-PD1 response. Finally, we identified that *cis*-regulatory loci of a co-receptor and negative regulator of TGF-β, *CD109* (Fig. 5f), and the nicotinic acetylcholine receptor *CHRNA1* (Fig. 5g), are specifically activated in response-associated T_ex_ cells, suggesting targets for future research.

### Revealing developmental history during cortical gyrification

**Fig. 6:**
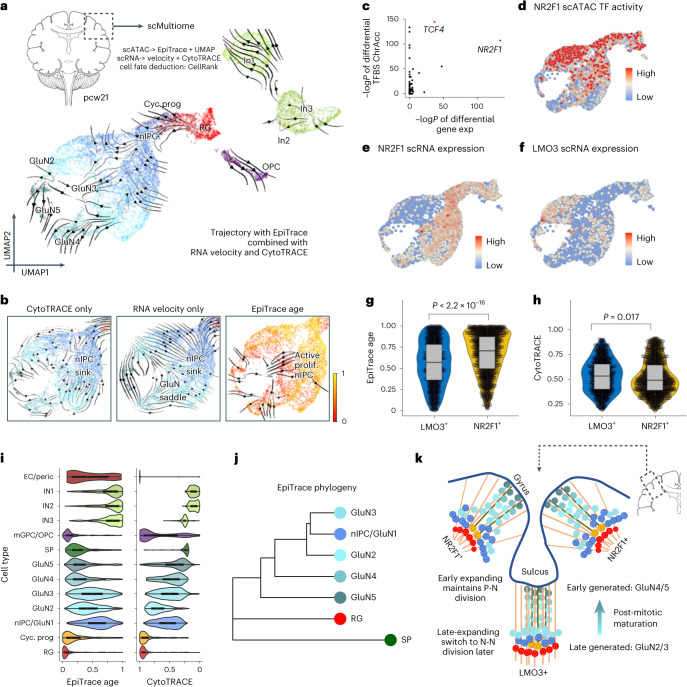
EpiTrace reveals the developmental history during human cortical gyrification. **a**, UMAP projected cell evolution trajectory built with CellRank by using a hybrid kernel of EpiTrace, CytoTRACE and RNA velocity of an scMultiomic-seq dataset from a pcw21 human brain. EC, endothelial cell; IN, inhibitory GABAergic neuron; mGPC/OPC, medial ganglionic eminence progenitor/oligodendrocyte precursor cell; SP, subplate neuron. SPs and ECs are not shown in the figure due to space limitations. **b**, Trajectories built with only CytoTRACE (i) or RNA velocity (ii) resulted in unrealistic ‘sinks’ and ‘saddles’ on the map. In contrast, EpiTrace age (iii) provided a unidirectional reference of time to reveal that the ‘sink’ nIPC population is mitotically active to resolve the ‘nIPC stall’. **c**, Scatter plot of the differential gene expression estimate (−log*P* value, *x* axis) and differential TFBS-specific ChrAcc estimate (−log*P* value, *y* axis) in the GluN cells. Most significantly differential expressed transcription factors NR2F1 and TCF4 are highlighted in the figure. Differential expression was estimated by non-parametric Wilcoxon rank-sum test. **d**, UMAP of TFBS-specific ChrAcc of NR2F1. **e**, Expression of *NR2F1* on UMAP. **f**, Expression of *LMO3* on UMAP. **g**, EpiTrace age of cells belong to the *LMO3*^+^ population or *NR2F1*^+^ population. Sample numbers of biologically independent cells: 808 (*LMO3*^+^) and 1,198 (*NR2F1*^+^). *P* < 2.2 × 10^−16^ (Wilcoxon test, two-sided; the *P* value resulted in numerical underflow). **h**, CytoTRACE of cells belong to the *LMO3*^+^ population or *NR2F1*^+^ population. Sample numbers as in **g**. *P* = 0.017 (Wilcoxon test, two-sided). **i**, Mitotic clock (EpiTrace) and differentiation potential (CytoTRACE) of the same cell in scMultiomic-seq. The CytoTRACE score was reversed to show differentiation from left to right to facilitate comparison with EpiTrace. Sample numbers of biologically independent cells: *n* = 646 (RG); 341 (Cyc. Prog.); 2,348 (nIPC/GluN1); 1,546 (GluN2); 798 (GluN3); 459 (GluN4); 223 (GluN5); 190 (SP); 359 (mGPC/OPC); 301 (IN3); 780 (IN2); 959 (IN1); and 31 (EC/Peric.). **j**, Excitatory neuron phylogeny built with mitotic clock, showing that GluN4/GluN5 are likely direct, early-born progenies of RG, whereas GluN2/GluN3 are likely late-born, immature progenies of nIPC. **k**, Overall model of corticogenesis in the light of EpiTrace. Data source: Trevino et al.^65^. For box plots, the upper and lower bounds of boxes show 25% and 75% percentiles of the data. The median of data is shown as the horizontal line in the box. The distribution minima and maxima, defined as farthest data point distanced ≤1.5 IQR from the box bounds, are shown by the whiskers. The violin plot shows the empirically estimated density distribution of data.

To test how mitotic age estimation might complement RNA-based development analysis, we applied EpiTrace to a scMultiomic dataset from the post contraception week (pcw) 21 human fetal brain cortex (GSE162170 (ref. ^65^)) to study the trajectory of glutaminergic neuron (GluN) development (Fig. 6a). GluNs develop from radial glia (RG) through the cycling progenitor (Cyc. Prog) cells into neuronal intermediate progenitor cells (nIPCs), before undergoing a cascade of maturation (GluN1 > GluN2 > GluN3 > GluN4 > GluN5)^65–67^. We modeled the cell fate transition by CellRank^68^ with kernels built with RNA velocity, CytoTRACE (an RNA-based index of cell differentiation state), EpiTrace age or a combined kernel with all three estimators. Although RNA velocity and CytoTRACE produced inconsistent transition trajectories that pointed toward a group of nIPCs (Fig. 6b, i and ii), kernels with EpiTrace age revealed a correct direction of development from the nIPCs toward terminally differentiated neurons (Fig. 6b, iii). The combined kernel of all three estimators resulted in a biologically plausible transition trajectory that starts from RG to bifurcate into two different branches, each giving rise to a distinct nIPC population that differentiates into mature neurons (Fig. 6a).

Two transcription factors, *TCF4* and *NR2F1* (encoding the transcription factor COUP-TFI), were differentially expressed between the branches. They exhibit significant differential binding activities in these neurons (Fig. 6c). Interestingly, *NR2F1* is mainly expressed in the gyrus of the human cortex, and hereditary *NR2F1* loss-of-function mutations are associated with mental retardation and the polymicrogyri phenotype^69–71^. NR2F1 TFBS-associated peaks are open in a branch (Fig. 6d) that is *NR2F1* negative (Fig. 6e) and *LMO3* positive (Fig. 6f), suggesting that NR2F1 turned into a transcriptional repressor in nIPCs. The EpiTrace age of the *NR2F1*^+^ branch nIPC was significantly higher than that of the *LMO3*^+^ nIPC, suggesting increased mitotic activity (Fig. 6g). In concordance with this, the CytoTRACE score of *NR2F1*^+^ nIPC was lower than that of LMO3^+^ nIPC (Fig. 6h), suggesting increased differentiation. These results indicate that nIPCs are divided into *NR2F1*^+^ clones that support earlier neurogenesis and *LMO3*^*+*^*/NR2F1*^*−*^ clones that expand relatively later, linking the gyrus-specific expression pattern of *NR2F1* to its function in cortical gyrification^69^.

We compared the EpiTrace age of the neurons with their CytoTRACE score (Fig. 6i). Although the CytoTRACE score of GluNs correlates with their differentiation, the EpiTrace age of these cells is inversely correlated with their maturity. To explain this inconsistency, we built a ‘phylogenetic tree’ of single cell clusters with ClockDML ChrAcc (EpiTrace phylogeny). We reasoned that cells traverse on the phenotype manifold on branched trajectories while they undergo mitosis. As they evolve, ChrAcc on ClockDML converges into a specific state that should be lineage dependent because of the irreversible nature of such change. Hence, it is possible to infer cell lineage trees using phylogenetic-like methods. Such analysis revealed a birth sequence of GluNs: GluN5 is first divided from RG, followed by GluN4, GluN2, GluN3 and GluN1/nIPC (Fig. 6j), indicating that neurons that formed earlier undergo longer post-mitotic maturation (Supplementary Fig. 27). In concordance with this observation, by analyzing scRNA expression of the same cells, we found that, whereas the late-aged nIPC/GluN1 and GluN2 cells still showed reminiscent RNA expression of the proliferating cells, such as *SOX11*, *SOX4*, *MALAT1* and *NFIB*, the earlier-aged, ‘more mature’ GluN5 and GluN4 cells showed significantly increased expression of mature neuron markers, including synaptic proteins, including *SYT4*, *SYT11*, *FABP7*, *APP*, *GAP43* and *PCDH17*; mature neuron cytoskeleton proteins, such as *TUBB2A* and *NEFL*; and post-mitotic functioning transcription factors, such as *MEF2C* (Supplementary Fig. 28). Furthermore, in concordance with the known ‘inside-out’ developmental paradigm of the cortex^72^, the earlier-aged GluN5 specifically expresses the layer V/VI marker genes *SCUBE1* and *SEMA3E*^73^, whereas the younger GluN4 population expresses similarly higher levels of the layer III/IV marker genes *NTNG1* and *MME*^73^ (Supplementary Fig. 29). Hence, the dynamics of post-mitotic neurons undergoing continuous differentiation could be captured by combining mitotic age with other modality measurements.

Together, this analysis demonstrated that EpiTrace age analysis complements RNA velocity and stemness prediction in characterizing complex organ development; indicated a long post-mitotic maturation of neurons; and revealed the molecular mechanism of *NR2F1* controlling human nIPC proliferation to underlie cortical gyrification (Fig. 6k).

### Inferring gene function in kidney from a static snapshot

**Extended Data Fig. 1:**
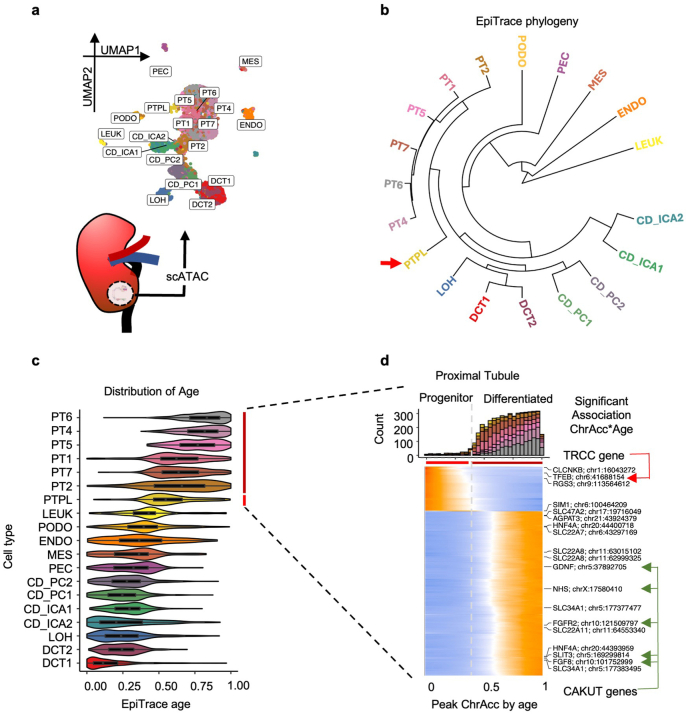
Single cell phylogeny of kidney from an adult tissue snapshot. **(a)** UMAP projection of scATAC data of normal human kidney samples from biopsies. Abbreviations: LEUK: leukocytes; ENDO: endothelial cells; MES: mesangial cells; PEC: parietal epithelial cells; PODO: podocytes; PT: proximal tubule; PTPL: proximal tubule progenitor-like cell; LOH: loop-of-Henri; DCT: distal collection tubule; CD_PC: collection duct principal cell; CD_ICA: collection duct intercalating cell. **(b)** Single cell phylogeny built with chromatin accessibility on ClockDML (EpiTrace phylogeny). **(c)** Distribution of EpiTrace age for each cell type. Sample number of biologically independent cells: n = 845 (DCT1); 408 (DCT2); 484 (LOH); 537 (CD_ICA2); 404 (CD_ICA1); 437 (CD_PC1); 472 (CD_PC2); 111 (PEC); 80 (MES); 245 (ENDO); 234 (PODO); 60 (LEUK); 204 (PTPL); 544 (PT2); 881 (PT7); 901 (PT1); 486 (PT5); 763 (PT4); 1055 (PT6). **(d)** Cell age distribution of PT cells (colored by type) (top) and ATAC peak activity across cell age (bottom) in PT cells. Genes of interest are labelled on the right. Known translocation renal cell carcinoma (TRCC) driver gene: *TFEB*; known hereditary renal dysgenesis (CAKUT) genes: *FGF8*, *FGFR2*, *SLIT3*, *GDNF*, and *NHS*. For boxplots, the upper and lower bounds of boxes show 25% and 75% percentile of the data. The median of data is shown as horizontal line in the box. The distribution minima and maxima, defined as farthest datapoint distanced <= 1.5IQR from the box bounds, were shown by the whiskers. Violin plot shows the empirically estimated density distribution of data.

We already demonstrated that EpiTrace can track development using developing tissues. To test whether EpiTrace can recover epigenomic changes during development from a single, terminally developed, static snapshot from adult tissue, we applied EpiTrace to an scATAC-seq dataset from adult human kidney (Extended Data Fig. 1a; GSE166547 (ref. ^74^)). The birth sequence of kidney cells by EpiTrace phylogeny analysis suggests an endothelial origin of kidney tubules and delineates a cell-type-specific generation cascade during nephrogenesis (Extended Data Fig. 1b), with correlation to their spatial position (Supplementary Fig. 30). The distribution of EpiTrace age for each cell type suggests a distal-to-proximal genesis cascade of nephron tubules with a late expansion of proximal tubules (PTs) (Extended Data Fig. 1c).

In the PT lineage, EpiTrace age-derived phylogeny could be orthogonally validated with small nuclear RNA (snRNA)-derived phylogeny (Supplementary Fig. 31; GSE121862 (ref. ^75^)). The correlation between EpiTrace age and peak openness showed clear segregation of peaks opened in progenitor or differentiated PT cells (Extended Data Fig. 1d). Notably, such association is not guided by known cell type information, indicating the power of EpiTrace in positioning single cells along their evolutionary trajectory. Interestingly, the translocation renal cell carcinoma (TRCC) driver gene *TFEB* is specifically activated in progenitor cells and shows an age-dependent decrease in activity. In contrast, all hereditary renal dysgenesis (CAKUT) genes, *FGF8*, *FGFR2*, *SLIT3*, *GDNF* and *NHS*, are associated with differentiated cell-specific, age-dependent increased peaks. These results suggest that CAKUT is linked to genes functioning in terminal PT cell fate determination and function, whereas TRCC oncogenesis is linked to the mis-expression of progenitor-specific transcription factors, possibly forcing the dedifferentiation of terminally differentiated PT cells into a stem-like state.

### Tracking glioblastoma clonal evolution

**Extended Data Fig. 2:**
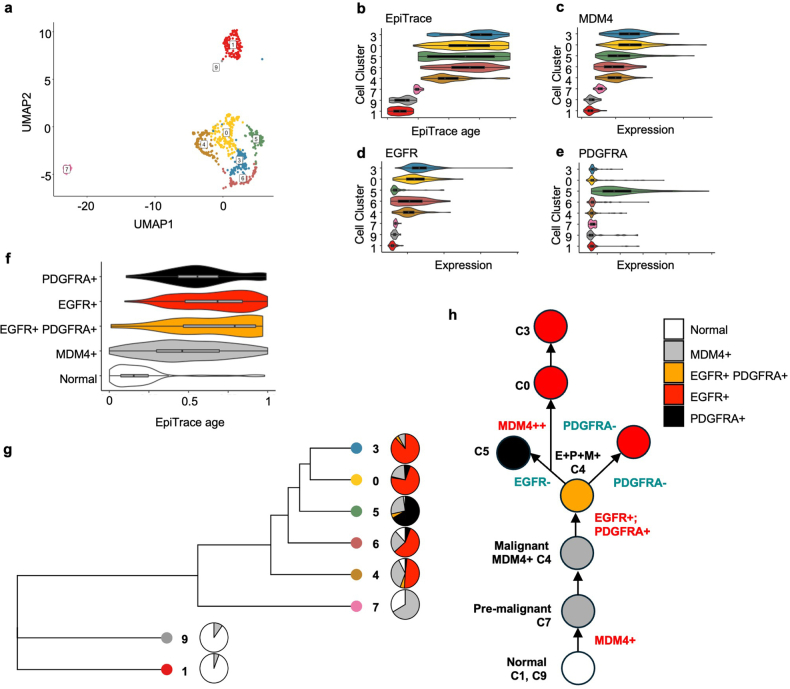
Early-on branching evolution of glioblastoma clonal evolution tracked by single cell mitotic age. **a)** UMAP of scATAC data from GBM sample CGY4349. Cell clusters are labeled (Numbers). Per-cell-cluster EpiTrace mitotic clock age **(b)** and *MDM4* **(c)**, *EGFR* **(d)**, *PDGFRA* **(e)** promoter ChrAcc. Sample number of biologically independent cells: n = 172 (group 0); 138 (group 1); 108 (group 3); 104 (group 4); 85 (group 5); 66 (group 6); 39 (group 7); 20 (group 9). Note that *MDM4* amplification is further gained in younger clones 0/3. **(f)** EpiTrace age of cells with normal karyotype, *MDM4*^+^, *MDM4*^+^/*EGFR*^+^/*PDGFRA*^+^, *MDM4*^+^/*EGFR*^+^, and *MDM4*^+^/*PDGFRA*^+^. **(g)** EpiTrace phylogeny, clonal composition (pie charts on the right), showing that the original pre-malignant cell cluster (7) evolved into an initial malignant clonal expansion with *MDM4* amplification, followed by a malignant *MDM4*^+^ population that was double-positive with *PDGFRA* and *EGFR* amplifications. In later stages, tumor clones split the oncogenic amplification and grow into either *PDGFRA*^+^ (5) or *EGFR*^+^ (6/0/3) populations. **(h)** Schematic evolution trajectory. For boxplots, the upper and lower bounds of boxes show 25% and 75% percentile of the data. The median of data is shown as horizontal line in the box. The distribution minima and maxima, defined as farthest datapoint distanced <= 1.5IQR from the box bounds, were shown by the whiskers. Violin plot shows the empirically estimated density distribution of data.

Finally, we analyzed an individual tumor sample (CGY2349) in a human glioblastoma (GBM) scATAC-seq dataset to study whether EpiTrace age analysis could work for cell evolution in oncogenesis (Extended Data Fig. 2a; GSE139136, GSE163655 and GSE163656 (ref. ^76^)). In this tumor, copy number variation (CNV) analysis showed that *MDM4* amplification dominates the malignant clones, which additionally have either *EGFR* or *PDGFRA* amplifications, resulting in increased ChrAcc around these genes (Extended Data Fig. 2b–e). With EpiTrace, we identified a pre-malignant cluster (7) that is younger than all malignant clones (4/6/5/0/3) but shows accelerated aging/mitosis count compared to the ‘normal clones’ (1/9) (Extended Data Fig. 2b,f), has lower *MDM4* amplification (Extended Data Fig. 2c) and is without either *EGFR* or *PDGFRA* amplification (Extended Data Fig. 2d–e).

Interestingly, some *MDM4*^+^ cells had both *EGFR* and *PDGFRA* amplification (Supplementary Fig. 32). EpiTrace age analysis revealed that the *MDM4*^+^-only cells are ancestral to triple-positive, *EGFR*^+^/*PDGFRA*^+^ cells, followed by loss of either *EGFR* or *PDGFRA* in the progeny (Extended Data Fig. 2f). This is further supported by EpiTrace phylogeny analysis (Extended Data Fig. 2g). Branched evolution of *MDM4*^*+*^*/EGFR*^*+*^ and *MDM4*^+^/*PDGFRA*^+^ cells was initiated at the beginning of malignant transformation (Supplementary Fig. 32). Together, these results characterized the evolutionary trajectory of malignancy from the *MDM4*^+^ pre-malignant clone to the earliest malignant cell population with amplification of *MDM4*, *PDGFRA* and *EGFR* in a catastrophic genomic instability event, which bifurcated into heterogeneous clones with either *PDGFRA* or *EGFR* addiction (Extended Data Fig. 2h). EpiTrace age analysis revealed the pre-malignant state of this tumor and suggested branching evolution of this tumor to indicate that heterogeneous cancer clones arise early in malignancy transformation.

It was previously known that telomere crisis and mitotic mis-segregation can cause catastrophic events in a single mitosis, most importantly chromothripsis^77,78^, chromoplexy^79^ and kataegis^80^. Multiple structural variations over the genome can occur simultaneously during such events, resulting in a synchronous, punctuated burst of chromosomal copy number aberration^77,81^. By timing the occurrence time of these mutational events, it was identified that such events occur early during oncogenesis^82,83^. *PDGFRA* and *EGFR* amplifications were reported to exist in different single-cell clones that coexist in a mosaic manner in GBM tumors^84^. Although most reports suggest that these mutations are mutually exclusive in single GBM-derived cell lines or tumor sphere cultures^85^, these clones coexist within the same tumor and share common somatic mutations, such as deletion of *PTEN* and *CDKN2A*^84,86^, indicating that they were derived from the same ancestral clone. scRNA-seq^87,88^ suggests that *PDGFRA*^+^/*EGFR*^+^ double-positive cells exist in GBM. Single-positive *PDGFRA*^+^ or *EGFR*^+^ descendent clones could emerge from double-positive parental clones without specific selection^86^. These observations are similar to our observation with EpiTrace. In our analysis, although we sampled only a fraction of the tumor, the similar cell age estimated for *MDM4*^*+*^*/EGFR*^*+*^ and *MDM4*^*+*^*/PDGFRA*^*+*^ clones suggested that neither of these clones gained selective advantage during tumor growth. Instead, they are under neutral evolution. Further experiments with higher-resolution clonal tracing, putatively with a genetic marker, are necessary to confirm this observation.

## Discussion

We formulated a model of clock-like ChrAcc change during cell mitotic aging, leading to the discovery of a universal epigenomic hallmark during cellular development: ChrAcc across clock-like loci. The heterogeneity of ChrAcc across clock-like loci is reduced at each mitosis, resulting in a converged, homogeneous activity pattern. We showed that ChrAcc changes act upstream of clock-like DNAm changes. Counting the fraction of opened clock-like loci of each cell gives a simple, phenotypic measure of cell mitotic age. We leveraged this measure to build a tool, called EpiTrace, to predict cellular mitotic age. Furthermore, we showed that the similarity across clock-like loci ChrAcc between single-cell clusters can serve as an accurate distance measure for phylogenetic analysis.

The DNAm shift in ClockDML is widely accepted as a hallmark of aging. However, the molecular mechanism generating age-dependent DNAm is yet unknown. Our data indicated that sample ages predicted by ChrAcc and DNAm were significantly correlated, suggesting that they are possibly under the control of a similar biological process.

ChrAcc changes accompany development and cell fate transition. However, we noticed that ChrAcc on clock-like loci is phenotypically neutral—that is, irrelevant to cell phenotype—based on several lines of evidence. First, EpiTrace age measured on the same set of clock-like loci generated from one tissue lineage (for example, the ClockDML from human PBMCs) works for different lineages. Second, the EpiTrace age of a single cell correlates with its accumulative mitosis number instead of developmental maturity (for example, in the case of neuronal development). Third, clock-like loci derived from one species could be used to predict single-cell age in another species. The exact molecular mechanism controlling how clock-like differential DNAm occurs on clock-like loci (to generate ClockDML), and how clock-like ChrAcc emerges on these loci, is an interesting question awaiting future investigation.

It is unexpected to us that the phylogenetic tree built with ChrAcc on clock-like loci (EpiTrace phylogeny) for single-cell clusters of the same developmental lineage is highly accurate. EpiTrace-inferred age is similar to pseudotime-inferred cell ‘developmental time’ but with higher resolution and less variation (Supplementary Fig. 33). In fact, we noticed that such phylogenetic trees sometimes outperform those built with the highly variable peaks from scATAC-seq data in terms of accuracy. Despite the fact that ClockDML (and clock-like loci) are highly enriched in *cis*-regulatory regions, there is no functional enrichment of them in specific developmental pathways or specific types of genomic elements (in addition to active *cis*-regulatory elements). Furthermore, this phenomenon is also phenotypically neutral. These results not only suggest a consistent birth sequence of cell types within the lineage but also indicate that senescence is a defined molecular process across cell types.

Our study is not without limitations. We noticed that the quality of inferred mitotic age by the current EpiTrace algorithm is dependent on sequencing depth (Supplementary Figs. 34–36). EpiTrace could be less accurate when working on cells with low sequencing depth. Additionally, EpiTrace could be inaccurate when the starting cell population is highly imbalanced (Supplementary Fig. 23). This phenomenon is related to single-cell population heterogeneity across development, the nature of ChrAcc shifts during mitotic aging, the statistical model underlying our algorithm and the limitations of the sequencing technique. Finally, the estimation accuracy and computational efficiency of EpiTrace rely heavily on the enrichment of clock-like loci in the initial reference loci set. In this view, the full set of scATAC-seq peaks or solo-WCGW sites, although they may contain clock-like loci, are not sufficiently enriched (Supplementary Fig. 37). As a result, inferring cell age from these reference loci is not as accurate as using the ClockDML set that we provided for the algorithm (Supplementary Fig. 38). Furthermore, using these loci as references is extremely computationally inefficient and renders single-cell dataset analysis virtually impossible. Future improvement of the algorithm might require in-depth study of molecular mechanisms driving age-dependent ChrAcc changes on clock-like loci, improvements in the algorithm to adapt with low-quality and highly imbalanced datasets and improvements in computational efficiency.

In conclusion, we showed mitosis-associated, age-dependent ChrAcc on clock-like loci, which usually harbor ClockDML. Based on this phenomenon, we developed computational method EpiTrace to track single-cell age using ChrAcc. By comparison studies, we showed that the ChrAcc-based mitosis age measure complements somatic mutation, RNA velocity and stemness predictions to predict the cell evolution trajectory with improved precision and power. We expect EpiTrace to be a useful tool for single-cell studies for delineation of cellular hierarchies and organismal aging.

## Methods

### Human and animal biospecimens

This study was conducted in accordance with the measures of the Declaration of Helsinki, local legislation and the ethics protocols of the Human Genetic Resource Preservation Center of Hubei Province, China (Hubei Biobank). For the human study, blood samples from healthy donors (total *n* = 71, including training *n* = 24 and validation *n* = 47) used in this study were pre-treated and preserved by the Hubei Biobank, approved by the institutional ethical review board (approval no. 2017038-1 and no. 2021125). Donors were of Chinese Han ethnicity, with an approximately 1:1 balanced sex ratio and aged 22–70 years. Informed consent was obtained from the donors and their guardians. For the mouse study, 74 sex-balanced C57BL/6 mice aged 7–403 d were used to collect tail tissue (including training *n* = 37 and validation *n* = 37), and pregnant C57BL/6 mice (*n* = 4, 4 months old, 13–14 d of pregnancy) were used to collect primary embryonic fibroblasts at Zhongnan Hospital of Wuhan University, which was approved by the institutional ethical review board (approval no. ZN2022246). The mice were purchased from WTLH Co., Ltd. All mice were kept under specific pathogen-free conditions in a temperature-controlled environment with a 12-h light/12-h dark cycle and with free access to food and water.

### Datasets used in this study

Datasets used are listed in Supplementary Table 2. Quality control basic statistics of the datasets from which raw data could be obtained are listed in Supplementary Table 2.

### Reference genomes

Original reference genomes used in this study are as follows: hg19, hg38 (GRCh38), mm10 (GRCm38.75), dm6, danRer10. FASTA sequences and genome synteny chain files were downloaded from UCSC (https://hgdownload.soe.ucsc.edu/downloads.html). R packages containing these reference genomes (BSgenome.Hsapiens.UCSC.hg19 version 1.4.3, BSgenome.Hsapiens.UCSC.hg38 version 1.4.5, BSgenome.Mmusculus.UCSC.mm10 version 1.4.3, BSgenome.Drerio.UCSC.danRer10 version 1.4.2 and BSgenome.Dmelanogaster.UCSC.dm6 version 1.4.1) were hosted by Bioconductor (https://www.bioconductor.org/). GRCh38-mm10 mix genomes were downloaded from the 10x Genomics website (https://www.10xgenomics.com/support/software/cell-ranger/downloads). Fly-to-human orthology mapping was downloaded from FlyBase (http://ftp.flybase.org/releases/FB2024_01/precomputed_files/orthologs/dmel_human_orthologs_disease_fb_2024_01.tsv.gz).

### Cell culture

Isolation of primary MEFs was performed as previously described^89^. In brief, embryonic day (E) 13–14 embryos were dissected from the uteri of pregnant C57BL/6 mice, separated from their yolk sac and homogenized with scissors in 0.25% trypsin-EDTA. Homogenized embryos were aspirated in DMEM supplemented with 10% FBS and 1% penicillin–streptomycin. Primary MEFs were maintained in the same medium. HEK293–dCas9–p300 cells were a gift from Yi Rao at Peking University. Immortalized MEF cells were a gift from Hui Jiang at the National Institute of Biological Sciences, Beijing. All immortalized cells were maintained in DMEM supplemented with 10% FBS and 1% penicillin–streptomycin.

### FACS of cultured cells into different cell cycle phases

For cell sorting, live MEFs at different passages were stained with Hoechst 33342 (5 μg ml^−1^, Invitrogen, H1399) at 37 °C for 20 min in the dark. After trypsin digestion, MEFs were resuspended in PBS with 1% FBS and collected in a sterile tube with a cell strainer cap (Falcon, 352235). Then, the MEFs were subjected to FACS. MEFs are first gated for whole cells and cell debris (forward scatter (FSC) and side scatter (SSC)), then for single cells (Horizon V450-A and Horizon V450-H) and, lastly, for cells in G1, S or G2/M phase according to DNA content. A BD FACSAria III sorter was used for cell sorting at the Flow Cytometry Core Facility of the Medical Research Institute, Wuhan University. BD FACSDiva (version 8.0.1) and FlowJo (version 7.6.2) software were used for sorting acquisition and analysis.

### sgRNA perturbation

sgRNA targeting ClockDML G8 set loci was designed manually (Supplementary Table 3). The sgRNAs were synthesized and packaged into lentivirus by GenScript. HEK293–dCas9–p300 cells were transduced with the lentivirus sgRNA library at a low multiplicity of infection (MOI) (~0.4). Puromycin was added to cells 2 d after transduction. Cells were collected for bulk bisulfite sequencing and RNA-seq. DNA and RNA were extracted from the cells using an AllPrep DNA/RNA dual-prep kit (Qiagen, 80204). The relative sgRNA expression was quality controlled by sequencing the polymerase chain reaction (PCR) product of the sgRNA barcode region.

### Single-stranded bisulfite capture sequencing

Genome-wide CpG bisulfite capture sequencing was performed as in Xiao et al.^41^ using the Tequila-7N protocol. In brief, genomic DNA was extracted from PBMCs, bisulfite converted, poly-dT tailed with terminal transferase, ligated with a poly-dA-extruded 3′ adaptor, linearly amplified for 12 cycles with a primer that complements the 3′ adaptor and ligated with a 5′ adaptor with seven random nucleotide overhangs. The resulting adaptor-ligated single-stranded inserts were amplified with Illumina P5/P7 sequencing adaptor primers and captured with the EpiGiant probe set (NimbleGen, 07138881001) following the manufacturer’s protocol. Post-capture libraries were retrieved, amplified, quantified and sequenced on an Illumina NovaSeq with PE150 format to 50 million reads.

### Multiplex anchored PCR (MArchPCR) bisulfite targeted sequencing

Mouse ClockDML CpG bisulfite sequencing was performed with the MArchPCR protocol. In brief, nested gene-specific primers targeting 347 ClockDML from the mouse MM285 methylation microarray (‘MM285 loci’)^48^ were designed by a machine-learning-assisted automated pipeline (to be described in a separate publication). Genomic DNA was extracted from PBMCs, bisulfite converted, poly-dT tailed with terminal transferase, ligated with a poly-dA-extruded 3′ adaptor and amplified by a set of 5′ gene-specific primer 1 (GSP1) targeting mouse MM285 loci and a 3′ common primer targeting the 3′ adaptor. A second round of amplification was performed with a set of 5′ gene-specific primer 2 (GSP2) targeting several base pairs 3′ of the GSP1 landing site and the same 3′ common primer. The resulting DNA library was then purified and re-amplified with Illumina P5/P7 sequencing adaptor primers. Libraries were retrieved, amplified, quantified and sequenced on the Illumina NovaSeq with PE150 format to 3 million reads.

### SHARE-seq on single cells

SHARE-seq was performed essentially following the original published protocol^90^ (https://www.protocols.io/workspaces/shareseq) with slight modifications to work on cryopreserved cells on MGI sequencers. Adaptor and barcode oligos were synthesized (Supplementary Table 4) by Sangon. Tn5 protein from Novoprotein (M045-01B) was assembled with customized mosaic end adaptor oligo in-house by mixing annealed adaptor oligo and the Tn5 protein at 1.5:1 molar ratio and incubating at room temperature for 30 min. Cryopreserved cells were thawed according to the 10x Genomics protocol with buffer exchange, fixed with formaldehyde at a 1% w/v final concentration for 10 min and quenched by glycine. Nuclei were isolated by using NIB buffer (10 mM Tris pH 7.5, 1 mM NaCl, 3 mM MgCl_2_, 0.1% Tween 20, 0.1% NP40, 0.01% digitonin, 0.75% w/v BSA) and washed with NIW buffer (NIB without NP40 and digitonin) twice. Tn5 tagmentation was performed with 25 pmol assembled Tn5 protein against 20,000 nuclei in tagmentation buffer (1× Tango buffer, 0.2% NP40, 32% DMF, supplemented with 0.008% digitonin, 0.08% Tween 20, 1.7% v/v NexGene RNase Inhibitor and a full Merck protease inhibitor cocktail tablet per 500 µl) for 30 min at 37 °C with shaking (500 r.p.m.) in a rotating incubator. Reverse transcription, hybridization with barcode oligos, ligation of the oligos, crosslinking pulldown, scATAC library amplification, cDNA amplification, re-transposition with the cDNA and scRNA library amplification were performed as described in the SHARE-seq V2 protocol, with our modified oligo set. Sample multiplexing was performed by splitting the round-1 oligo barcode usage. In all experiments, 4–6 cell samples were multiplexed for each SHARE-seq preparation. DNA nanoballs were prepared with the MGI nanoball MDA kit following the producer’s protocol. Sequencing was performed on an MGI2000 sequencer with a modified cycle setting and customized primers. Sequencing setup was as follows: 101 bp from Tn5 transposed 5′ end using TN5-read1-link1/TN5-read1-link2 primers, 8 + 8 bp (with 30-bp dark cycle) using APP-A barcode primer 2, MDA reaction, 99 bp from Tn5 transposed 3′ end using Tn5-read2 primer and 8 bp using R1-oligo-R2-LK-RP primer. Sequencing depth was targeted to 500 million reads for a full SHARE-seq scATAC or scRNA library.

### Pre-processing of sequencing data

All bisulfite sequencing (methylation) data were processed exactly as described in Xiao et al. ^41^. Per-CpG DNA methylation frequency (beta) was computed with PileOMeth (version 0.1.13-3-gca82747, https://github.com/bgruening/PileOMeth). All bulk ATAC and CUT&RUN sequencing data were processed with the bulk ATAC processing procedure described in Xiao et al.^41^, with the hg19 reference genome. The 10x scATAC sequencing data were processed by CellRanger-ATAC (version 1.2.0) with the hg38 reference genome. 10x scMultiomic sequencing data were processed by CellRanger-ARC (version 2.0.1) with the hg38 reference genome. 10x scRNA data were aligned to the hg38 reference transcriptome by kallisto (version 0.46.1, https://github.com/pachterlab/kallisto) and converted to a splice/unsplice count matrix. SHARE-seq data were processed separately for the scRNA and scATAC datasets. SHARE-seq ATAC sequencing data were pre-processed by zUMI (2.9.7b) to barcode-corrected ubam, converted to FASTQ by SAMtools and mapped to the GRCh38-mm10 hybrid genome (10x Genomics) or mm10 genome by bwa (0.7.17-r1188). Cell barcodes were extracted by sinto (version 0.9.0) using the ‘nametotag’ command, and fragments were extracted by the ‘fragment’ command. SHARE-seq scRNA data were processed by StarSolo (version 2.7.10a_alpha_220818) using splice junction database (sjdb) with a 50-bp overhang and the key parameters ‘–soloCBposition 0_0 0_7 0_8_0_15 0_115_0_122 \–soloUMIposition 0_18_0_25–soloCellFilter CellRanger2.2 8000 0.99 10 \–soloCBmatchWLtype 1MM \–outFilterMultimapNmax 1 \–outFilterScoreMinOverLread 0.1 \–outFilterMatchNminOverLread 0.1’. The output velocyto result was then used in downstream processing. Fragment files from these pre-processed ATAC data were combined and analyzed in ArchR (version 1.0.1)^6^. Quality control, doublet removal, dimensionality reduction (latent semantic indexing (LSI)), clustering and peak finding were all performed in ArchR according to its reference. Annotation of cells from their original publications/dataset was performed without adjustment. Cells were not manually excluded from the analysis except in three cases: (1) 3PN cells in the human embryonic development dataset, which were aneuploid and expected to behave differently compared to the karyotypically normal cells; (2) PT3 cluster in the kidney scATAC dataset, which is donor specific; and (3) CD34-500 erythroid lineage in the in vitro CD34 differentiation dataset, of which the EpiTrace age estimation is aberrant due to imbalanced cell composition. Lift-over between reference genome sets was executed with easyLift (version 0.2.1, https://github.com/caleblareau/easyLift). scRNA data were passed to Seurat^91^ (version 4) for basic quality control, normalization, scaling, dimensionality reduction (principal component analysis (PCA)) and clustering. The Seurat object was converted to anndata format with SeuratObjects (version 4.0.4) before RNA velocity and CytoTRACE analysis. For ATAC and CUT&Tag data processing, the reads were aligned to hg19 reference genome using bwa and de-duplicated by sambamba (version 0.5.4) before peak calling by MACS2 (2.2.7.1).

### Age-dependent clock-like DML

Age-dependent, clock-like DML (chronology) were discovered in the training cohort, with correlating per-CpG beta values against sample donor age using an adapted code from scAge (version 1.0.0, Trapp et al.^31^, and personal communication with Alexandre Trapp). The cutoff of the absolute value of Pearson’s correlation between sample age and beta was set to 0.7. Known clock-like mitosis-associated DML from Yang et al.^28^ (mitosis) and development-associated DML from Zhou et al.^38^ (solo-WCGW) were also included for comparison. Enrichment of clock-like DML on *cis*-regulatory elements from human scATAC^40^, hematopoiesis cell ATAC^42^, bladder cancer scATAC^41^ and placenta scATAC (Gong et al., unpublished) datasets was performed with Fisher’s exact test in R. Clock-like DML were clustered according to their beta and sample age using hclust in R (4.1.3), and unsupervised classification was performed by cutree. The mean beta of each group of Clock-like DML was correlated with age. The group (G8) of clock-like DML with the best negative correlation between mean beta and sample age is shown in Fig. 1b.

### Predicting sample age by DNAm

Age prediction by DNAm beta values was performed by either GLM trained according to the method of Horvath^25^ or a modified scAge code (TimeSeq model)^92^. The training cohort did not include donors aged 0–18 years; thus, no log transformation was performed for younger donors. For the GLM, the loci were filtered for sufficient coverage >30×. We performed PCA on the CpG DNAm matrix and selected only PC1 for age prediction. For the TimeSeq model, the loci were not filtered.

### Measuring the diversity of ChrAcc in the ClockDML region

ATAC peaks overlapping ClockDML were pulled with GenomicRanges findOverlaps, and peakwise counts, intersample peak means, medians, standard deviations and coefficients of variation (CVs) were calculated in the R (version 4.1.3) package sparseMatrixStats (version 1.6.0). The entropy of ClockDML ChrAcc was measured by first normalizing per-peak read counts to total counts over all peaks of interest to have a vector of probability of observing a read in peak *x* : p(*x*), and computing the Shannon entropy as: Entropy = −1 × sum(p(*x*) × log(p(*x*))).

### Measuring the general accessibility of the ClockDML region

EpiTrace measures the total accessibility of given reference (ClockDML, or clock-like loci) region. ATAC peaks overlapping ClockDML were pulled with GenomicRanges findOverlaps, and peakwise counts were optionally censor-normalized according to Qiu et al.^93^. ClockAcc, defined as the number of total opened DML, was measured for each cell for peaks with non-zero read coverage. The algorithm then performed cell‒cell similarity-based reaction-diffusion smoothing to leverage information from other cells to denoise ClockAcc. To do this, cell‒cell similarity was first calculated. The inter-single-cell dispersion of each peak was calculated and used to select the top 5% of variable peaks. The cell‒cell similarity (correlation) matrix was computed as the correlation of the top 5% of variable peaks between each single cell using WGCNA::cor (version 1.70-3). The correlation matrix was further thresholded by its mean, with correlation values lower than the mean being set to zero. Then, the correlation vector for each cell was normalized by dividing the total sum of correlation, denoting the cell‒cell similarity index. Assuming the mitotic age acts on the phenotype of cell (manifested as a cell‒cell similarity matrix) to result in smoothened ClockAcc, the mitotic age of cells could be solved by non-negative least squares (NNLS). NNLS decomposition of the cell‒cell similarity matrix and smoothed ClockAcc was computed by the Lawson‒Hanson algorithm using nnls::nnls (version 1.4). Reaction-diffusion regression of the NNLS result was performed using an HMM-like approach: in each iteration, the NNLS result is updated to the weighted sum of the current NNLS result and the cross-product of the NNLS result with the cell‒cell similarity matrix until the difference between the updated NNLS result and the current NNLS result is lower than the preset threshold. The cross-product of the final NNLS result with the cell‒cell similarity matrix results in a smoothed, regressed measure of ClockAcc. The overall algorithm was first proposed in CytoTRACE^3^. The resulting smoothed total ChrAcc was renormalized between 0 and 1 to give a rank of samples; such rank denotes the relative birthday (mitotic age) of the cell within the population. For bulk sequencing, the rank is reversed as (1-rank).

### Iterative algorithm to measure the general accessibility of the ClockDML region in scATAC data

Single-cell ATAC data are under-sequenced and sparse. Hence, reads falling on known reference clock-like loci (or ClockDML) might be few for individual cells. We use correlation to pick additional genomic loci whose ChrAcc strongly correlates with the initial estimated cell age, and then we use them as additional ‘reference clock-like loci’ to boost the algorithm performance. In brief, after the re-normalization of smoothed total ChrAcc, the rank is not reversed. For iteration, read counts of all ATAC peaks were correlated against single-cell age estimation (the rank). The correlation coefficient was scaled, and peaks with a sufficiently high correlation coefficient versus age (normally Z > 2.5 or 3) were selected. A new ‘reference clock-like loci’ set was built as the union of high correlation peaks and the previous reference clock-like loci. In the next round of iteration, this new ‘reference clock-like loci’ set replaces the previous reference for computation. The iteration was performed for designated times or until convergence of age estimation. Final single-cell EpiTrace age estimation was given by the rank of smoothed total ChrAcc over clock-like loci in the final iteration. No reversal of ranking was performed for single-cell datasets.

### Phylogenetic tree construction with ChrAcc in the ClockDML region

A mitosis birth sequence-based phylogenetic tree was inferred with ChrAcc on ClockDML in Seurat with the function BuildClusterTree and rooted using the package ‘ape’ (version 5.6-1) with the cell cluster that has minimal mean EpiTrace age serving as an outgroup.

### Association of ChrAcc with estimated cell age

The read counts of all ATAC peaks were correlated against sample age estimation with the R (version 4.1.3) package WGCNA and BiocParallel (verrsion 1.28.3). Correlation coefficients were scaled by R. Age-dependent ChrAcc trends of loci with significant correlations were analyzed with tradeSeq (version 1.8.0). To generate a heatmap for visualization, ChrAcc of the same locus was averaged for cells of similar age bin (0–100) and phenotype.

### Uniform manifold approximation and projection and cell transition trajectory for embryonic ATAC data

The ATAC data were collected from public databases (PRJNA494280 (ref. ^19^) and PRJNA394846 (ref. ^45^)) and in-house placenta ATAC data. After alignment, peaks were called for each data point and merged to become a union ATAC peak set. The union peak set was then used as the input feature set. A read count matrix (peak × cell, 253,541 × 44) was constructed and normalized by Signac::RunTFIDF(). A total of 250,290 variable features were identified by Signac::FindTopFeatures (min.cutoff = 50). Dimensional reduction was performed with Signac::RunSVD() and uniform manifold approximation and projection (UMAP) by Seurat::RunUMAP (reduction = ‘lsi’, dims = 3:6). A pseudotime trajectory was constructed using Slingshot, and an arrow was drawn according to the smoothed trajectory.

### Cross-mapping of ClockDML between species

When genome synteny lift-over chains are available (human–mouse, human–zebrafish, mouse–human and mouse–zebrafish), lift-over between genomes for the ClockDML was performed with UCSC LiftOver executable with the respective chain file. For fly–human mapping, without available lift-over chains, ortholog pairs were identified bioinformatically using the FlyBase database. Human orthologous genes with ClockDML present in their promoter were identified. Then, the corresponding promoters in the fly ortholog genes were taken as ‘human-guided’ fly clock-like loci.

### Comparing eight-cell-like cell–development cluster hierarchy inference results with known results

We first built a ‘real cluster hierarchy’ based on RNA expression of known four-cell-like (4CL) (*TET1/NCOA3/FGFR1/HYAL4/WNT3/DNMT3L/LEFTY1/DHCR24*) and eight-cell-like cell (8CLC) (*DPPA3/ZNF280A/TPRX1/ZSCAN4/DUXA)* marker genes, extracellular matrix markers (*FN1/COL1A1/COL3A1/EMP1/CDH11*) and the cluster-specific differentially expressed genes. The clusters were individually ranked according to EpiTrace age and scVelo pseudotime, and each rank was subsequently correlated with the ‘real cluster hierarchy’.

### Mitochondrial SNV (mGATK) analysis

mGATK (version 0.6.1) analyses were performed following Lareau et al.^8^ to reproduce the clones described in the original publication.

### Clonal age inference and expansion ratio estimation

For each mitochondrial (mt) scATAC cell myeloid-biased clone from the CD34_800 experiment, the clone was first classified by whether it harbored only progenitor (prog/prog_my) cells (Prog-only), intermediate (my_1) cells (Int-only) or both. The difference in the total numbers of terminal cells (my2, my3 and my4) in this clone on latter timepoints (D14/D20) from that on the initial timepoint (D8) was taken as the ‘expanded cell number’. The mean EpiTrace age of cells on the initial timepoint (D8) was taken as the ‘initial age of this clone’. Finally, the log-expanded cell number was regressed against the initial age for each clone.

### RNA velocity and CytoTRACE analysis

Spliced and unspliced scRNA data were exported from R (version 4.1.3) with SeuratObjects to h5ad format and imported into Python (version 3.9.7) to be processed by CellRank (version 1.5.1, https://github.com/theislab/cellrank)^68^. The scVelo and CellRank CytoTRACE kernels were used for estimating RNA velocity and CytoTRACE score, respectively. scVelo (version 0.2.5, https://github.com/theislab/scvelo)^94^ with Scanpy (1.9.3) was used to produce velocity embeddings in UMAP space.

### Multiomic derivation of cell evolution trajectory with kernel combining cell age and RNA velocity

A custom kernel of CellRank built upon EpiTrace age was used to project EpiTrace age into CellRank. A combined kernel of RNA velocity and EpiTrace was built with 0.75 × velocity + 0.25 × age, which was used in the downstream Generalized Perron Cluster Cluster Analysis (GPCCA) estimator in CellRank. Lineages were calculated by GPCCA, with cell clusters with the least age designated root and cell clusters with the maximal age designated terminal. Velocity genes were computed by the GPCCA estimator for a given lineage.

### Transcription factor motif differential analysis

TFBS motif differential activity was determined by Signac (version 1.5.0, https://github.com/timoast/signac)^95^ using the same EpiTrace object built during EpiTrace analysis.

### CNV analysis

scATAC CNV was called with CopySCAT (version 0.3.0, https://github.com/spcdot/CopyscAT)^76^ and annotated with ChIPseeker (version 1.30.3)^96^.

### Long-read nanoNOME-seq data analysis

Processed methylated C calling data were downloaded from the National Center for Biotechnology Information (NCBI) (GSE183760 (ref. ^55^)). Methylation calls were performed to call a base ‘methylated’ with log_lik_ratio > 1 or ‘unmethylated’ with log_lik_ratio < −1. All bases with log_lik_ratio between −1 and 1 were called ‘unknown’. Nucleosome-sized (147-bp) flanks were taken from each ClockDML site, and the CpG and GpC methylation states were called for each CpG and GpC site within this region. A region is defined as ‘opened’ if the fraction of ‘methylated’ GpC exceeds 20%. For pseudotime analysis, methylation-per-base information of the reads from the same Cas9-targeted region was used to construct a matrix, which was then used by TSCAN (1.34.0) to compute a pseudotime evolution sequence of the reads.

### scCOOL NOME-seq data analysis

Sequencing data from human embryonic cell scCOOL-seq experiments^43^ were mapped to the hg19 reference genome using bwa-meth (0.2.0). Per-read CpG and GpC co-classified epireads were extracted using PileOMeth (0.1.13-3-gca82747 using HTSlib version 1.2.1). Reads overlapping the ESC mitosis-related EpiTOC^28^ loci were extracted. For each stage of cells, the mean CpG and GpC methylation levels on these reads were calculated and summarized.

### Bisulfite conversion PCR data processing and correlation to the human ClockDML aging coefficient

The bisulfite conversion PCR (bsPCR) data from PRJNA490128 (ref. ^56^) were downloaded from the NCBI and mapped to the GRCm38.75 genome with bwa-meth. The CpG methylation levels were extracted with PileOMeth. The sequenced CpG loci were mapped back to the human genome using the mm10-hg19 chain using LiftOver. Transactivation-mediated DNAm shifts in the mouse loci were then compared to the human age-dependent DNAm shift coefficient.

### Pseudotime trajectory inference (Slingshot, VIA, Monocle and Palantir)

Slingshot (2.2.0), VIA (pyVIA 0.1.77), Monocle2 (2.22.0), Monocle3 (0.2.0) and Palantir (1.3.1) computations of the mtscATAC dataset were performed exactly as the software instructions mentioned.

### GO set enrichment

Genes from the clustering result of the anti-PD1 scATAC dataset (C1, C2 and C3) were used for GO enrichment analysis using the R package clusterProfiler^97^ (4.4.4) with the function ‘enrichGO’. Pathways with adjusted *P* values less than 0.05 were considered significant.

### Region overlap enrichment

Overlap enrichment between regions was tested by Fisher’s exact test. For random permutation, background random loci were permuted for indicated (100 or 1,000) times using regioneR (1.28.0).

### Statistics and visualization

Grouped comparisons between individual data points were performed with two-sided Student’s *t*-test or Wilcoxon test where applicable. One-sided Fisher’s exact test was used for gene set enrichment analysis. Multiple comparison adjustment was performed for the GO analysis. Two-sided Fisher’s exact test was performed for loci-to-loci enrichment analysis. For correlation analysis, correlation coefficient (*R*) and *P* value were calculated with Pearson’s method. For linear or LOESS regression, 95% confidence intervals (CIs) are shown as shades over the regressed line. For box and whisker plots of grouped data points, the upper and lower bounds of boxes show 25% and 75% percentiles of the data. The median of data is shown as the horizontal line in the box. The distribution minima and maxima, defined as farthest data point distanced ≤1.5 interquartile range (IQR) from the box bounds, are shown by the whiskers. The violin plot shows the empirically estimated density distribution of data. To plot enrichment analysis results from Fisher’s exact test, odds ratio is shown as the median point in the graph, and its 95% CI is shown as error bars.

### Reporting summary

Further information on research design is available in the Nature Portfolio Reporting Summary linked to this article.

## Online content

Any methods, additional references, Nature Portfolio reporting summaries, source data, extended data, supplementary information, acknowledgements, peer review information; details of author contributions and competing interests; and statements of data and code availability are available at 10.1038/s41587-024-02241-z.

## Supplementary information

Supplementary Notes, Supplementary Figs. 1–38, brief description of Supplementary Tables 1–4, Supplementary Table 5 and References for Supplementary Information.

Reporting Summary

Supplementary Table 1 Human ClockDML identified in this study.

Supplementary Table 2 Datasets used in this study and quality control basic statistics for datasets with raw data available.

Supplementary Table 3 sgRNA designed for the ClockDML G8 set.

Supplementary Table 4 Oligos used for SHARE-seq on the MGI platform.

## Data Availability

A full list of downloaded and generated data used in this study, including their access IDs, references, usage in this paper and/or download links, is provided in Supplementary Table 2. Accession codes for the publicly available datasets used in this study are as follows: (National Center for Biotechnology Information (NCBI)): PRJNA494280; PRJNA394846; GSE178969; GSE190130; GSE178324; GSE178966; GSE142745; GSE129785; GSE162170; GSE166547; GSE139136; GSE163655; GSE163656; GSE74912; GSE89895; GSE179606; GSE65360; GSE163579; GSE137115; GSE152423; GSE164978; GSE100272; GSE183760; PRJNA522707; GSE102395; GSE103590; and GSE121862. (China National GeneBank DataBase (CNGB)): CNP0001454. Chemical induction of eight-cell-like state data was downloaded from https://figshare.com/s/ff707bf8242f7b3ed8f5, https://figshare.com/s/760d3ff54f1214a50cc2 and https://figshare.com/s/9c01c3b58d34b80de230. mtscATAC data were downloaded from https://github.com/caleblareau/mtscATACpaper_reproducibility. anti-PD1-treated cancer biopsy data were downloaded from https://github.com/GreenleafLab/MPAL-Single-Cell-2019. Cortical scMultiomic data were downloaded from https://github.com/GreenleafLab/brainchromatin. In-house-generated datasets were uploaded to the OMIX database in the CNGB (https://ngdc.cncb.ac.cn/omix/): human data: OMIX005823 (ref. ^98^) and mouse/in vitro data: OMIX005824 (ref. ^99^). No restrictions will be applied on using the in-house-generated mouse and in vitro datasets. Due to local legal requirements, the in-house-generated human dataset will be available only in processed data format and will require a case-by-case application through the China Human Genetic Resources Management Office. Contact Kaiyu Qian, Wuhan University, via the CNGB OMIX website (‘Request for this data’) for the access. The data manager will respond within 1 week.

## Code availability

EpiTrace and accompanying testing data are available on GitHub^100^. Tutorials and usage manuals for EpiTrace are provided on readthedocs^101^. Codes to reproduce the analysis, along with the original and processed data for reproducibility purposes, are provided on OSF^102^.
