# Supplementary material for: Tracking single-cell evolution using clock-like chromatin accessibility loci
Source: Nat Biotechnol. 2024 May 9;43(5):784–98. doi: 10.1038/s41587-024-02241-z (PMC12084158; doi:10.1038/s41587-024-02241-z)
Supplement: Supplementary file 2 — Reporting Summary [file 41587_2024_2241_MOESM2_ESM.pdf]

nature portfolio

Corresponding author(s): Xinghuan Wang, Yi Zhang

Last updated by author(s): Apr 9, 2024

Reporting Summary

Nature Portfolio wishes to improve the reproducibility of the work that we publish. This form provides structure for consistency and transparency in reporting. For further information on Nature Portfolio policies, see our Editorial Policies and the Editorial Policy Checklist.

Statistics

For all statistical analyses, confirm that the following items are present in the figure legend, table legend, main text, or Methods section.

n/a	Confirmed	
☐	☒	The exact sample size (*n*) for each experimental group/condition, given as a discrete number and unit of measurement
☐	☒	A statement on whether measurements were taken from distinct samples or whether the same sample was measured repeatedly
☐	☒	The statistical test(s) used AND whether they are one- or two-sided<br>*Only common tests should be described solely by name; describe more complex techniques in the Methods section.*
☐	☒	A description of all covariates tested
☐	☒	A description of any assumptions or corrections, such as tests of normality and adjustment for multiple comparisons
☐	☒	A full description of the statistical parameters including central tendency (e.g. means) or other basic estimates (e.g. regression coefficient) AND variation (e.g. standard deviation) or associated estimates of uncertainty (e.g. confidence intervals)
☐	☒	For null hypothesis testing, the test statistic (e.g. *F*, *t*, *r*) with confidence intervals, effect sizes, degrees of freedom and *P* value noted<br>*Give P values as exact values whenever suitable.*
☒	☐	For Bayesian analysis, information on the choice of priors and Markov chain Monte Carlo settings
☒	☐	For hierarchical and complex designs, identification of the appropriate level for tests and full reporting of outcomes
☐	☒	Estimates of effect sizes (e.g. Cohen's *d*, Pearson's *r*), indicating how they were calculated

Our web collection on statistics for biologists contains articles on many of the points above.

Software and code

Policy information about availability of computer code

Data collection	Publicly available data were collected from public repositories and websites (see Supplementary Dataset 2). Genome-wide CpG bisulfite capture sequencing was performed using the Tequila-7N protocol, captured with the EpiGiant probe set (Nimblegen, Cat. #07138881001), and sequenced on an Illumina NovaSeq with PE150 format to 50 M reads. Mouse ClockDML CpG bisulfite sequencing was performed with the MArchPCR protocol and sequenced on an Illumina NovaSeq with PE150 format to 3 M reads. SHARE-seq was performed essentially following the original published protocol and sequenced to 500 M reads per a full Shareseq scATAC or scRNA library on an MGI MGI-2000 sequencer.
Data analysis	The R environment used throughout the study was v4.1.3. The Python environment used throughout the study was v3.9.7. Bisulfite sequencing data were aligned to the hg19 reference genome using bwa-meth (0.2.0). Per-CpG DNA methylation frequency (beta) was computed with PileOMeth (0.1.13-3). Age dependent, clock-like DMLs were inferred by scAge (v1.0.0). All bulk ATAC and Cut-And-Run sequencing data were aligned to the hg19 reference genome using bwa (0.7.17-r1188) and de-duplicated by sambamba (v0.5.4). Peak calling was performed with MACS2 (2.2.7.1). The 10× scATAC sequencing data were processed by CellRanger-ATAC (v1.2.0) with the hg38 reference genome. 10x scMultiomic sequencing data were processed by CellRanger-ARC (v2.0.1) with the hg38 reference genome. 10x scRNA data were aligned to the hg38 reference transcriptome by kallisto (v0.46.1) and converted to a splice/unsplice count matrix. SHARE-seq data were processed separately for the scRNA and scATAC datasets. SHARE-seq ATAC sequencing data were preprocessed by zUMI (2.9.7b) and mapped to the GRCh38-mm10 hybrid genome (10x Genomics) or mm10 genome by bwa. Fragments were generated by sinto (0.9.0). SHARE-seq scRNA data were processed by StarSolo (version 2.7.10a_alpha_220818). Fragment files from preprocessed ATAC data were combined and analyzed in ArchR (v1.0.1). Lift-over between reference genome sets was executed with easyLift (v0.2.1). scRNA data were processed by Seurat (v4). SeuratObjects (v4.0.4) was used for anndata conversion. Scanpy (1.9.3), scVelo (0.2.5) and CellRank (1.5.1) were used for RNA velocity, group trajectory, and stemness (CytoTRACE) analysis. For age estimation using scATAC, data were analyzed with the developed package, EpiTrace (v0.0.0.9000), which is available at https://github.com/MagpiePKU/EpiTrace. The software is provided as a complete R package that runs under R (4.1.3). Successful installation of the package makes a reproducible software environment. Example data (bulk FACS-sorted blood cell ATAC sequencing) were provided with the code as test data. mGATK (v0.6.1) was used for mitochondrial mutation calling from scATAC. Signac (v1.5.0) was used for TFBS activity inference from scATAC. CopySCAT (v0.3.0) was used for CNV calling from scATAC. ChIPseeker (v1.30.3) was used for peak annotation. TSCAN (1.34.0) was used for pseudotime analysis for long-read NanoNOME.

clusterProfiler (4.4.4) was used for pathway enrichment analysis. regioneR (1.28.0) was used for peak enrichment analysis. Pseudotime inference used Slingshot (2.2.0), VIA (pyVIA 0.1.77), Monocle2 (2.22.0), Monocle3 (0.2.0), and Palantir (1.3.1). Additional R packages used in the study are: sparseMatrixStats (v1.6.0), WGCNA (v1.70-3), nnls (v1.4), ape (v5.6-1), tradeSeq (v1.8.0), BiocParallel (v1.28.3). BD FACSDiva (v8.0.1) and FlowJo (v7.6.2) were used for sorting acquisition and analysis.

For manuscripts utilizing custom algorithms or software that are central to the research but not yet described in published literature, software must be made available to editors and reviewers. We strongly encourage code deposition in a community repository (e.g. GitHub). See the Nature Portfolio guidelines for submitting code & software for further information.

Data

Policy information about availability of data

All manuscripts must include a data availability statement. This statement should provide the following information, where applicable:

- Accession codes, unique identifiers, or web links for publicly available datasets
- A description of any restrictions on data availability
- For clinical datasets or third party data, please ensure that the statement adheres to our policy

Tutorials and usage manuals for EpiTrace are provided on publicly accessible website (https://epitrace.readthedocs.io). Codes to reproduce the analysis, along with the original and processed data for reproducibility purpose, are provided on OSF (https://osf.io/8xd2p, doi:10.17605/OSF.IO/8XD2P), which is publicly accessible.

In-house generated datasets are uploaded to OMIX database of CNGB, China (https://ngdc.cncb.ac.cn/omix/): OMIX005823 (human data) and OMIX005824 (mouse and in vitro data). No restrictions would be applied on using the in-house generated mouse and in vitro datasets. Due to local legal requirements, the in-house generated human dataset would only be available in processed data format and requires a case-by-case application through the China Human Genetic Resources Management Office. Please contact through the CNGB OMIX website ("Request for this data") to contact Dr. Kaiyu Qian, Wuhan University for the access. The data manager would response within a week.

Accession codes for the publicly available datasets in this study are: (NCBI): PRJNA494280; PRJNA394846; GSE178969; GSE190130; GSE178324; GSE178966; GSE142745; GSE129785; GSE162170; GSE166547; GSE139136; GSE163655; GSE163656; GSE74912; GSE89895; GSE179606; GSE65360; GSE163579; GSE137115; GSE152423; GSE164978; GSE100272; GSE183760; PRJNA522707; GSE102395; GSE103590 ; GSE121862. (CNGB): CNP0001454. Chemical induction of 8-cell-like state data is downloaded from https://figshare.com/s/ff707bf8242f7b3ed8f5, https://figshare.com/s/760d3ff54f1214a50cc2 and https://figshare.com/s/9c01c3b58d34b80de230. mtscATAC data is downloaded from https://github.com/caleblareau/mtscATACpaper_reproducibility. anti-PD1 treated cancer biopsy data is downloaded from https://github.com/GreenleafLab/MPAL-Single-Cell-2019. Cortical scMultiome data downloaded is from https://github.com/GreenleafLab/brainchromatin.

Original reference genomes used in this study are: hg19, hg38 (GRCh38), mm10 (GRCm38.75), dm6, danRer10. FASTA sequences and genome synteny chain files were downloaded from UCSC (https://hgdownload.soe.ucsc.edu/downloads.html). R packages containing these reference genomes (BSgenome.Hsapiens.UCSC.hg19 v1.4.3, BSgenome.Hsapiens.UCSC.hg38 v1.4.5, BSgenome.Mmusculus.UCSC.mm10 v1.4.3, BSgenome.Drerio.UCSC.danRer10 v1.4.2, BSgenome.Dmelanogaster.UCSC.dm6 v1.4.1) were hosted by Bioconductor (https://www.bioconductor.org/). GRCh38-mm10 mix genome were downloaded from 10x Genomics website (https://www.10xgenomics.com/support/software/cell-ranger/downloads). Fly-to-human orthology mapping is downloaded from FlyBase (http://ftp.flybase.org/releases/FB2024_01/precomputed_files/orthologs/dmel_human_orthologs_disease_fb_2024_01.tsv.gz).

Field-specific reporting

Please select the one below that is the best fit for your research. If you are not sure, read the appropriate sections before making your selection.

☒ Life sciences ☐ Behavioural & social sciences ☐ Ecological, evolutionary & environmental sciences

For a reference copy of the document with all sections, see nature.com/documents/nr-reporting-summary-flat.pdf

Life sciences study design

All studies must disclose on these points even when the disclosure is negative.

Sample size	No sample size for single cell study is pre-determined for this analysis. For aging-associated ClockDML analysis, the samples were selected such that they evenly represent different age bins and within each bin there were at least three individuals.
Data exclusions	Single cells that are excluded from the study were: 1. 3PN embryo scATAC data, since they were aneuploid and were expected to behave differently compared to the karyotypically normal samples; 2. PT3 cells from the kidney dataset, which is donor-specific, and 3. erythroid lineage cells from the CD34_500 in vitro differentiation dataset. The exclusion criteria was determined according to common biology knowledge. The exclusion of CD34_500 erythroid lineage cells were because their cell type were highly imbalanced. The effect of cell type imbalance in this dataset was analyzed and shown in Supplemental Figure 23.
Replication	The reproducibility of the results were done by: 1. analyzing sub-sampled datasets; 2. reproducing the result on different computers with different operating systems (Linux and Mac); and 3. reproducing the result by three individuals by running the code at different hardware and software environments. Each experiment has been replicated by at least three times. The replication was always successful in producing qualitatively similar results.
Randomization	Complete randomization is performed for segregating the training and validation sample cohort to discover genome-wide DML.
Blinding	All computational methods were blinded to ground truth cell type labels/pairing information, development hierarchy, or sample donor age during performance evaluation.

Reporting for specific materials, systems and methods

We require information from authors about some types of materials, experimental systems and methods used in many studies. Here, indicate whether each material, system or method listed is relevant to your study. If you are not sure if a list item applies to your research, read the appropriate section before selecting a response.

Materials & experimental systems

n/a	Involved in the study	
☒	☐	Antibodies
☐	☒	Eukaryotic cell lines
☒	☐	Palaeontology and archaeology
☐	☒	Animals and other organisms
☐	☒	Human research participants
☒	☐	Clinical data
☒	☐	Dual use research of concern

Methods

n/a	Involved in the study	
☒	☐	ChIP-seq
☐	☒	Flow cytometry
☒	☐	MRI-based neuroimaging

Eukaryotic cell lines

Policy information about cell lines

Cell line source(s)	HEK293-dCas9-p300 (Dr. Yi Rao, Peking University, China); MEF (Dr. Hui Jiang, National Institute of Biological Sciences, China)
Authentication	The HEK293-Cas9-p300 line was authenticated by PCR of the transgene. MEF was authenticated by comparing the scRNA/ scATAC sequencing result to those from known MEF cells.
Mycoplasma contamination	The cell lines were not tested for mycoplasma contamination.
Commonly misidentified lines (See ICLAC register)	No commonly misidentified cell lines were used in the study.

Animals and other organisms

Policy information about studies involving animals; ARRIVE guidelines recommended for reporting animal research

Laboratory animals	The wild-type C57BL/6 mice (1:1 balanced sex, age between 7 days to 403 days) and pregnant female C57BL/6 mice (4 months old, 13-14 days of pregnancy) were purchased from WTLH Co., Ltd., (Beijing, China). All mice were kept under specific pathogen free (SPF) and temperature-controlled environment with 12h light/12h dark cycle, and free access to food and water.
Wild animals	No wild animals were used in the study.
Field-collected samples	No field collected samples were used in the study.
Ethics oversight	The animal protocol was approved by the Institutional Ethical Review Board of Zhongnan Hospital of Wuhan University (approval number: ZN2022246).

Note that full information on the approval of the study protocol must also be provided in the manuscript.

Human research participants

Policy information about studies involving human research participants

Population characteristics	Healthy Chinese Han ethnicity donors with 1:1 balanced sex ratio and aged between 22-70 years old. The donors were recruited from in-campus volunteer advertisement. We did not note any self-selection bias or other biases that may be present in donor recruitment process.
Recruitment	This study was conducted in accordance with the measures of the Declaration of Helsinki and the Ethic Protocols of Human Genetic Resource Preservation Center of Hubei Province, China (Hubei Biobank). Blood samples from healthy donors (total n=71) were used in this study.
Ethics oversight	Blood samples from healthy adult donors used in this study were pretreated and preserved by the Hubei Biobank, approved by the Institutional Ethical Review Board (approval number: 2017038-1 and 2021125). Informed consent was obtained from the donors and their guardians.

Note that full information on the approval of the study protocol must also be provided in the manuscript.

Flow Cytometry

Plots

Confirm that:

☒ The axis labels state the marker and fluorochrome used (e.g. CD4-FITC).

☒ The axis scales are clearly visible. Include numbers along axes only for bottom left plot of group (a 'group' is an analysis of identical markers).

☒ All plots are contour plots with outliers or pseudocolor plots.

☒ A numerical value for number of cells or percentage (with statistics) is provided.

Methodology

Sample preparation	Primary MEFs were collected from E13-14 embryos. Embryos were dissected from the uteri of pregnant C57BL/6 mice, separated from their yolk sac, and homogenized with scissors in 0.25% trypsin-EDTA. Homogenized embryos were aspirated in DMEM supplemented with 10% FBS and 1% penicillin and streptomycin. Primary MEFs were maintained in the same medium, and cultured to designated passage. Live MEFs at different passages were stained with Hoechst 33342 (5 µg/mL, Invitrogen, H1399) at 37°C for 20 min in the dark. After trypsin digestion, MEFs were resuspended in PBS with 1% FBS and collected in a sterile tube with a cell-strainer cap (Falcon, 352235). Then, the MEFs were subjected to FACS sorting.
Instrument	BD FACSAria III cell sorter
Software	BD FACSDiva (v8.0.1) and FlowJo (v7.6.2) were used for sorting acquisition and analysis.
Cell population abundance	Targeted populations were sorted with single-cell precision.
Gating strategy	MEFs are first gated for whole cells and cell debris (FSC and SSC), then for single cells (Horizon V450-A and Horizon V450-H), and lastly for cells in G1, S, or G2/M phase according to DNA content.

☒ Tick this box to confirm that a figure exemplifying the gating strategy is provided in the Supplementary Information.
